# Bone Status in a Mouse Model of Experimental Autoimmune-Orchitis

**DOI:** 10.3390/ijms22157858

**Published:** 2021-07-23

**Authors:** Fabian Hemm, Monika Fijak, Jan Belikan, Marian Kampschulte, Thaqif El Khassawna, Adrian Pilatz, Christian Heiss, Katrin Susanne Lips

**Affiliations:** 1 Department of Trauma, Hand and Reconstructive Surgery, University Hospital of Giessen, Rudolf-Buchheim-Str. 7, 35392 Giessen, Germany; christian.heiss@chiru.med.uni-giessen.de; 2 Experimental Trauma Surgery, Justus-Liebig-University Giessen, Aulweg 128, 35392 Giessen, Germany; thaqif.elkhassawna@chiru.med.uni-giessen.de; 3 Department of Anatomy and Cell Biology, Justus-Liebig-University Giessen, Aulweg 123, 35385 Giessen, Germany; monika.fijak@anatomie.med.uni-giessen.de; 4 Laboratory of Experimental Radiology, Justus-Liebig-University Giessen, Schubertstrasse 81, 35392 Giessen, Germany; jan.d.belikan@radiol.med.uni-giessen.de (J.B.); marian.kampschulte@radiol.med.uni-giessen.de (M.K.); 5 Department of Urology, Pediatric Urology and Andrology, University Hospital of Giessen, Rudolf-Buchheim-Straße 7, 35392 Giessen, Germany; adrian.pilatz@chiru.med.uni-giessen.de

**Keywords:** experimental autoimmune-orchitis, osteoporosis, mouse model, micro-CT, biomechanical properties, testosterone, osteocalcin

## Abstract

Investigations in male patients with fertility disorders revealed a greater risk of osteoporosis. The rodent model of experimental autoimmune-orchitis (EAO) was established to analyze the underlying mechanisms of male infertility and causes of reduced testosterone concentration. Hence, we investigated the impact of testicular dysfunction in EAO on bone status. Male mice were immunized with testicular homogenate in adjuvant to induce EAO (*n* = 5). Age-matched mice were treated with adjuvant alone (adjuvant, *n* = 6) or remained untreated (control, *n* = 7). Fifty days after the first immunization specimens were harvested. Real-time reverse transcription-PCR indicated decreased bone metabolism by alkaline phosphatase and Cathepsin K as well as remodeling of cell-contacts by Connexin-43. Micro computed tomography demonstrated a loss of bone mass and mineralization. These findings were supported by histomorphometric results. Additionally, biomechanical properties of femora in a three-point bending test were significantly altered. In summary, the present study illustrates the induction of osteoporosis in the investigated mouse model. However, results suggest that the major effects on bone status were mainly caused by the complete Freund’s adjuvant rather than the autoimmune-orchitis itself. Therefore, the benefit of the EAO model to transfer laboratory findings regarding bone metabolism in context with orchitis into a clinical application is limited.

## 1. Introduction

Testicular inflammation is mainly caused by bacterial infections, either sexually transmitted or from the urinary tract, and often develops a chronic asymptomatic disease progression [1,2,3,4]. Therefore, orchitis can persist over prolonged periods until adequate therapy is started and may result in irreversible long-term damage regarding the reproductive system [2,3]. In this regard, inflammatory infiltrates in the testicular interstitium, damaged seminiferous tubules and disorder of spermatogenesis have been illustrated [2]. In most cases inflammation affects epididymides as well, and leads to a combined epididymo-orchitis [1,2]. Consequently, orchitis, or rather epididymo-orchitis, represents a frequent cause of fertility disorders in young men [3,5]. However, the majority of fertility disorders are diagnosed a long time after the initial inflammatory process. Thus, in vitro and in vivo models are crucial to investigate the underlying processes of epididymo- orchitis and its complications. In this context the rodent model of experimental autoimmune-orchitis (EAO) represents a well-established animal model to evaluate the processes of testicular inflammation, consequences regarding the reproductive functionality and possible therapy options [6,7,8,9]. Furthermore, EAO was found to significantly reduce the testosterone concentration in rats in a similar way to human orchitis [6]. Androgens are known to represent a major impact factor regarding bone metabolism in men [10,11,12]. Accordingly, human studies illustrated the development of osteoporosis even in young men suffering from hypogonadism [13,14]. Therefore, we assumed EAO might also influence bone metabolism in the sense of causing osteoporosis, and consequently investigated bone status in a mouse model of EAO as there were no previous studies available.

## 2. Results

### 2.1. Histomorphometric Bone Architecture

**Figure 1:**
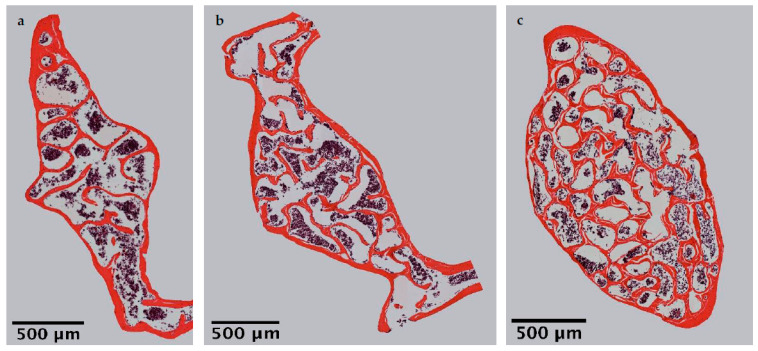
Microscopic overviews of vertebral bodies L3 stained with hematoxylin-eosin. Images taken at 40× magnification were merged. Mice were immunized with (**a**) testicular homogenate in adjuvant (EAO), (**b**) adjuvant alone or (**c**) remained untreated.

**Figure 2:**
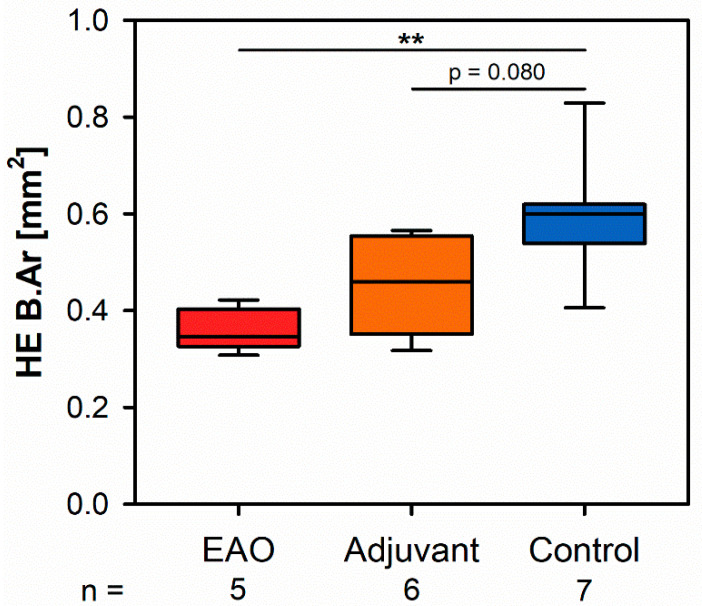
Histomorphometrical results regarding the bone area (B.Ar) in vertebral bodies L3 stained with hematoxylin-eosin (HE). Mice were immunized with testicular homogenate in adjuvant (EAO), adjuvant alone (adjuvant) or remained untreated (control). ** *p* < 0.01.

Microscopic overviews of hematoxylin-eosin-stained vertebral bodies (Figure 1) were automatically evaluated regarding bone area (B.Ar), trabecular thickness (Tb.Th) and trabecular separation (Tb.Sp), while the trabecular perimeter (Tb.Pm) was measured manually. Histomorphometrical measurement illustrated a significantly decreased bone area due to EAO (0.361 ± 0.020 mm^2^) and nearly significant reduction in the adjuvant group (Adj; 0.453 ± 0.043 mm^2^) compared to the control group (Cont; 0.594 ± 0.048 mm^2^, *p* < 0.01 vs. EAO, *p* = 0.080 vs. Adj, Figure 2). However, trabecular perimeter (EAO 17.082 ± 1.190 mm, Adj 17.358 ± 0.658 mm, Cont 21.550 ± 2.274 mm), trabecular thickness (EAO 17.050 ± 1.942 µm, Adj 17.509 ± 0.904 µm, Cont 18.024 ± 0.971 µm) and trabecular separation (EAO 106.298 ± 12.552 µm, Adj 108.196 ± 3.266 µm, Cont 87.265 ± 6.338 µm) remained without significant alterations (Figure S1). Detailed results are shown in Table S1.

### 2.2. Microarchitecture of Cortical Bone

**Figure 3:**
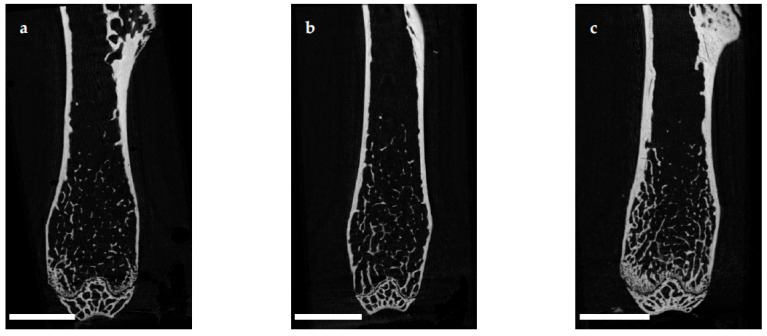
Two-dimensional images from µCT scans of distal femora (scale bar 2 mm). Mice were immunized with (**a**) testicular homogenate in adjuvant (EAO), (**b**) adjuvant alone or (**c**) remained untreated.

The microarchitecture of the femoral cortex was analyzed at mid diaphysis and distal diaphysis by micro computed tomography (µCT). Two-dimensional example images from µCT scans (Figure 3) demonstrated a loss of cortical as well as trabecular bone mass following (a) EAO or (b) treatment with adjuvant alone, compared to (c) untreated mice.

**Figure 4:**
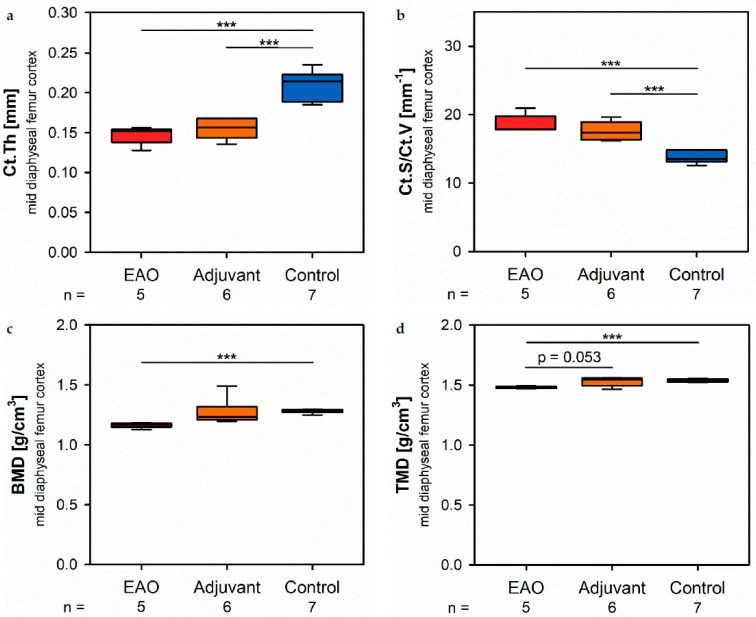
Microarchitecture of mid diaphyseal femur cortex in µCT. Measurement of (**a**) cortical thickness (Ct.Th), (**b**) cortical surface/volume ratio (Ct.S/Ct.V), (**c**) bone mineral density (BMD) and (**d**) tissue mineral density (TMD). Mice were immunized with testicular homogenate in adjuvant (EAO), adjuvant alone (adjuvant) or remained untreated (control). *** *p* < 0.001.

At the mid diaphyseal region, µCT identified a significantly reduced cortical thickness (Ct.Th, Figure 4a) in the EAO (0.147 ± 0.005 mm) and adjuvant group (0.155 ± 0.005 mm) in contrast to the control group (0.210 ± 0.007 mm, *p* < 0.001 each) with no difference between EAO and adjuvant. The cortical surface (Ct.S, not graphically displayed) remained unaltered in EAO (10.538 ± 0.163 mm^2^) compared to control (10.270 ± 0.235 mm^2^, *p* = 1.000), but slightly decreased in the adjuvant group (9.530 ± 0.238 mm^2^, *p* < 0.05 vs. EAO, *p* = 0.087 vs. Cont). In contrast, the cortical surface/volume ratio (Ct.S/Ct.V, Figure 4b) was significantly increased in EAO (18.588 ± 0.608 mm^−1^) and adjuvant (17.594 ± 0.549 mm^−1^) compared to control (13.670 ± 0.326 mm^−1^, *p* < 0.001 each). Therefore, the cortical surface/volume ratio indirectly indicated a loss of cortical bone volume in EAO and adjuvant without significant differences between them. The bone mineral density (BMD, Figure 4c) was reduced in EAO (1.164 ± 0.010 g/cm^3^, *p* < 0.001 vs. Cont 1.280 ± 0.006 g/cm^3^) while adjuvant (1.270 ± 0.045 g/cm^3^) showed no difference to control. Similarly, the tissue mineral density (TMD, Figure 4d) was decreased in the EAO group (1.480 ± 0.004 g/cm^3^) compared to control (1.537 ± 0.005 g/cm^3^, *p* < 0.001) and almost significantly decreased compared to the adjuvant group (1.531 ± 0.015 g/cm^3^, *p* = 0.053 vs. EAO) with no difference between adjuvant and control (*p* = 0.974). Detailed results are shown in Table S2.

**Figure 5:**
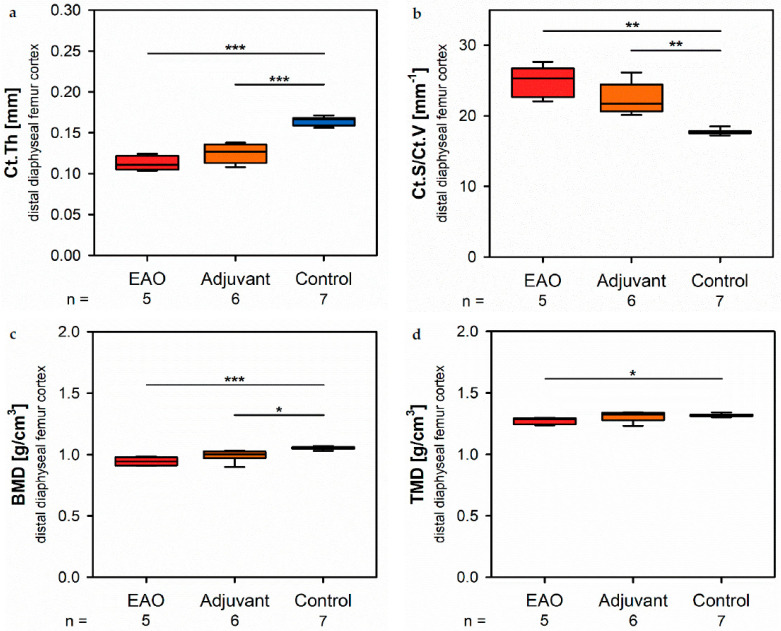
Microarchitecture of distal diaphyseal femur cortex in µCT. Measurement of (**a**) cortical thickness (Ct.Th), (**b**) cortical surface/volume ratio (Ct.S/Ct.V), (**c**) bone mineral density (BMD) and (**d**) tissue mineral density (TMD). Mice were immunized with testicular homogenate in adjuvant (EAO), adjuvant alone (adjuvant) or remained untreated (control). * *p* < 0.05; ** *p* < 0.01; *** *p* < 0.001.

At the distal diaphysis, µCT showed a significantly thinner cortical thickness (Figure 5a) in the EAO (0.113 ± 0.004 mm) and adjuvant group (0.125 ± 0.005 mm) compared to the control group (0.165 ± 0.002 mm, *p* < 0.001 each) similar to the mid diaphysis. The cortical surface (not graphically displayed) remained without differences in all groups (EAO 13.012 ± 0.274 mm^2^, Adj 11.556 ± 0.326 mm^2^, Cont 12.686 ± 0.438 mm^2^). Again, the cortical surface/volume ratio (Figure 5b) was significantly increased in the EAO (24.713 ± 0.982 mm^−1^) and adjuvant group (22.396 ± 0.915 mm^−1^) in contrast to control (17.706 ± 0.152 mm^−1^, *p* < 0.01 each) and indicated a loss of cortical bone volume indirectly. The bone mineral density (Figure 5c) was reduced in EAO (0.944 ± 0.015 g/cm^3^) compared to control (1.055 ± 0.005 g/cm^3^, *p* < 0.001). Additionally, bone mineral density at distal diaphysis was also decreased in the adjuvant group (0.993 ± 0.020 g/cm^3^, *p* < 0.05) unlike at the mid diaphysis. In contrast, the tissue mineral density (Figure 5d) was only reduced in the EAO group (1.273 ± 0.012 g/cm^3^) compared to control (1.319 ± 0.005 g/cm^3^, *p* < 0.05) while adjuvant remained unaltered (1.309 ± 0.017 g/cm^3^, *p* = 1.000). Detailed results are shown in Table S3.

### 2.3. Microarchitecture of Trabecular Bone

**Figure 6:**
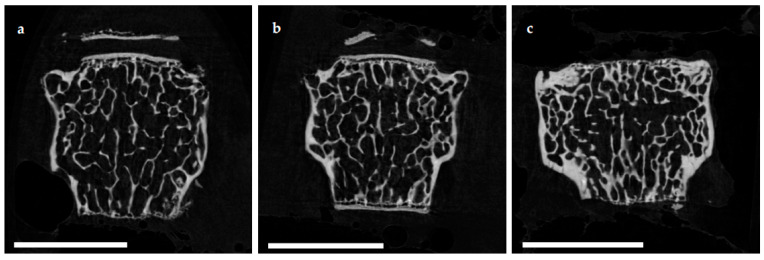
Two-dimensional images from µCT scans of vertebral bodies L1 (scale bar 2 mm). Mice were immunized with (**a**) testicular homogenate in adjuvant (EAO), (**b**) adjuvant alone or (**c**) remained untreated.

The microarchitecture of trabecular bone was studied in femora and lumbar vertebral bodies (L1) by µCT. Two-dimensional example images from µCT scans of vertebral bodies (Figure 6) illustrate a loss of trabecular bone mass following (a) EAO or (b) treatment with adjuvant alone compared to (c) untreated mice, similar to µCT scans of the distal femora (Figure 3).

**Figure 7:**
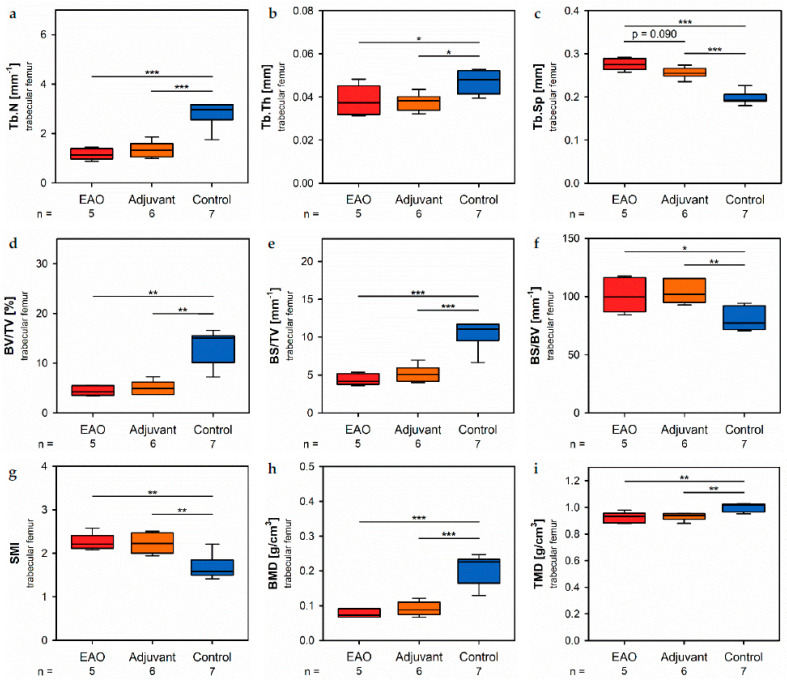
Microarchitecture of trabecular femur by µCT. Measurement of (**a**) trabecular number (Tb.N), (**b**) trabecular thickness (Tb.Th), (**c**) trabecular separation (Tb.Sp), (**d**) bone volume fraction (BV/TV), (**e**) bone surface density (BS/TV), (**f**) specific bone surface (BS/BV), (**g**) structure model index (SMI), (**h**) bone mineral density (BMD) and (**i**) tissue mineral density (TMD). Mice were immunized with testicular homogenate in adjuvant (EAO), adjuvant alone (adjuvant) or remained untreated (control). * *p* < 0.05; ** *p* < 0.01; *** *p* < 0.001.

In the trabecular region of the femora, µCT indicated a loss of trabecular number (Tb.N, Figure 7a) and trabecular thickness (Tb.Th, Figure 7b) in EAO (Tb.N: 1.171 ± 0.099 mm^−1^; Tb.Th: 0.038 ± 0.003 mm) and in the adjuvant group (Tb.N: 1.344 ± 0.133 mm^−1^; Tb.Th: 0.038 ± 0.002 mm) compared to control (Tb.N: 2.771 ± 0.197 mm^−1^, *p* < 0.001 each; Tb.Th: 0.048 ± 0.002 mm, *p* < 0.05 each). Trabecular separation (Tb.Sp, Figure 7c) was larger in EAO (0.276 ± 0.006 mm) and adjuvant (0.256 ± 0.005 mm) than in control (0.199 ± 0.006 mm, *p* < 0.001 each). Consequently, the bone volume fraction (BV/TV, Figure 7d), as well as the bone surface density (BS/TV, Figure 7e) were significantly decreased in EAO (BV/TV: 4.452 ± 0.447%; BS/TV: 4.427 ± 0.335 mm^−1^) and adjuvant groups (BV/TV: 5.065 ± 0.566%; BS/TV: 5.171 ± 0.440 mm^−1^) in comparison to control (BV/TV: 13.340 ± 1.283%, *p* < 0.01 each; BS/TV: 10.340 ± 0.698 mm^−1^, *p* < 0.001 each). Thereby, the specific bone surface (BS/BV, Figure 7f) increased in the EAO (101.214 ± 6.639 mm^−1^, *p* < 0.05 vs. Cont 79.430 ± 3.681 mm^−1^) and adjuvant groups (103.878 ± 4.293 mm^−1^, *p* < 0.01). This finding illustrates a pronounced loss of trabecular bone volume over trabecular bone surface. Furthermore, the structure model index (SMI; Figure 7g) and trabecular pattern factor (Tb.Pf, not graphically displayed) were significantly elevated in EAO (SMI: 2.252 ± 0.087; Tb.Pf: 37.822 ± 2.271 mm^−1^) and adjuvant (SMI: 2.231 ± 0.100; Tb.Pf: 38.736 ± 2.703 mm^−1^) compared to control (SMI: 1.699 ± 0.102, *p* < 0.01 each; Tb.Pf: 22.782 ± 2.360 mm^−1^, *p* < 0.01 each), both indicating a degradation of trabecular integrity. The bone mineral density (BMD, Figure 7h) was reduced in both immunized groups (EAO 0.078 ± 0.006 g/cm^3^, Adj 0.091 ± 0.008 g/cm^3^) in contrast to control (0.205 ± 0.016 g/cm^3^, *p* < 0.001 each) similar to the femoral cortex. Equally, the tissue mineral density (TMD, Figure 7i) was decreased in both immunized groups (EAO 0.922 ± 0.018 g/cm^3^, Adj 0.931 ± 0.012 g/cm^3^) compared to control (1.002 ± 0.012 g/cm^3^, *p* < 0.01 each). Detailed results are shown in Table S4.

**Figure 8:**
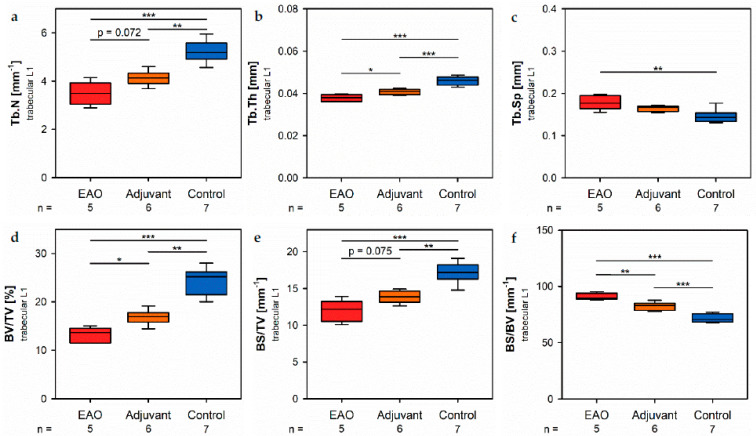

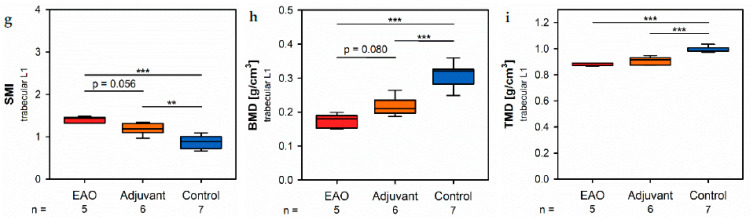
Microarchitecture of trabecular vertebral body L1 by µCT. Measurement of (**a**) trabecular number (Tb.N), (**b**) trabecular thickness (Tb.Th), (**c**) trabecular separation (Tb.Sp), (**d**) bone volume fraction (BV/TV), (**e**) bone surface density (BS/TV), (**f**) specific bone surface (BS/BV), (**g**) structure model index (SMI), (**h**) bone mineral density (BMD) and (**i**) tissue mineral density (TMD). Mice were immunized with testicular homogenate in adjuvant (EAO), adjuvant alone (adjuvant) or remained untreated (control). * *p* < 0.05; ** *p* < 0.01; *** *p* < 0.001.

Investigation of the trabecular region in vertebral bodies L1 by µCT (Figure 8) demonstrated generally similar alterations like the trabecular region in femora (Figure 7). However, several significant differences between EAO and the adjuvant group were detected in the trabecular region of vertebral bodies L1, while both groups were mostly comparable in the trabecular region of the femora.

In the vertebral bodies, L1 trabecular number (Figure 8a) and trabecular thickness (Figure 8b) were significantly decreased in the EAO (Tb.N: 3.488 ± 0.215 mm^−1^; Tb.Th: 0.038 ± 0.001 mm) and adjuvant groups (Tb.N: 4.131 ± 0.123 mm^−1^; Tb.Th: 0.041 ± 0.001 mm) compared to control (Tb.N: 5.243 ± 0.176 mm^−1^, *p* < 0.001 vs. EAO, *p* < 0.01 vs. Adj; Tb.Th: 0.046 ± 0.001 mm, *p* < 0.001 each). Furthermore, trabecular number was nearly (*p* = 0.072) and trabecular thickness significantly (*p* < 0.05) lower in EAO than in the adjuvant group. The trabecular separation (Figure 8c) was larger following EAO (0.179 ± 0.008 mm) than in the control group (0.147 ± 0.006 mm, *p* < 0.01), but unaltered in the adjuvant group (0.164 ± 0.003 mm, *p* = 0.113). These findings resulted in a significant reduction of the bone volume fraction (Figure 8d) as well as the bone surface density (Figure 8e) in EAO (BV/TV: 13.154 ± 0.711%; BS/TV: 11.951 ± 0.659 mm^−1^) and in the adjuvant group (BV/TV: 16.856 ± 0.628%; BS/TV: 13.868 ± 0.342 mm^−1^) in comparison to control (BV/TV: 24.056 ± 1.132%, *p* < 0.001 vs. EAO, *p* < 0.01 vs. Adj; BS/TV: 17.149 ± 0.535 mm^−1^, *p* < 0.001 vs. EAO, *p* < 0.01 vs. Adj). Bone volume fraction was significantly lower (*p* < 0.05), and bone surface density was almost lower (*p* = 0.075) in the EAO than in the adjuvant group. The specific bone surface (Figure 8f) was increased in the adjuvant group (82.504 ± 1.489 mm^−1^, *p* < 0.001 vs. Cont 71.647 ± 1.474 mm^−1^) and even more significantly raised after EAO (90.882 ± 1.376 mm^−1^, *p* < 0.001 vs. Cont, *p* < 0.01 vs. Adj), again indicating a predominant loss of trabecular bone volume over trabecular bone surface. Both the structure model index (Figure 8g) and trabecular pattern factor (not graphically displayed) illustrated a loss of trabecular integrity in EAO (SMI: 1.400 ± 0.033; Tb.Pf: 21.194 ± 0.562 mm^−1^) and adjuvant groups (SMI: 1.188 ± 0.054; Tb.Pf: 16.390 ± 1.011 mm^−1^) compared to control (SMI: 0.876 ± 0.061, *p* < 0.001 vs. EAO, *p* < 0.01 vs. Adj; Tb.Pf: 10.544 ± 0.927 mm^−1^, *p* < 0.001 vs. EAO, *p* < 0.01 vs. Adj). Again, EAO was more severely affected in this regard than the adjuvant group (SMI: *p* = 0.056; Tb.Pf: *p* < 0.01). The bone mineral density (Figure 8h) was decreased in both immunized groups (EAO 0.173 ± 0.009 g/cm^3^, Adj 0.216 ± 0.011 g/cm^3^) in contrast to control (0.308 ± 0.014 g/cm^3^, *p* < 0.001 each). Likewise, the tissue mineral density (Figure 8i) was reduced in EAO (0.883 ± 0.004 g/cm^3^) and adjuvant groups (0.908 ± 0.012 g/cm^3^) compared to control (0.995 ± 0.008 g/cm^3^, *p* < 0.001 each). Detailed results are shown in Table S5.

### 2.4. Osteoblasts

**Figure 9:**
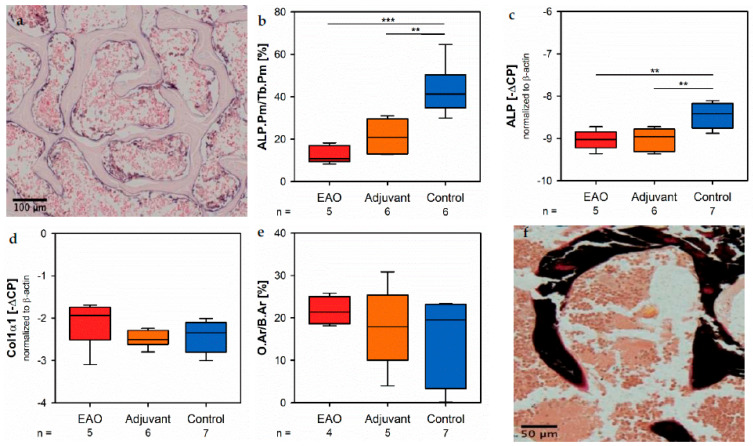
Osteoblasts. (**a**) Enzyme histochemical staining of alkaline phosphatase (ALP, violet) in vertebral body L3 from an untreated mouse. (**b**) Histomorphometrical calculation of relative ALP perimeter (ALP.Pm/Tb.Pm) in enzyme histochemical-stained vertebral bodies L3. mRNA expression of (**c**) ALP and (**d**) collagen 1α1 (Col1α1) in real-time RT-PCR of vertebral bodies Th10. (**e**) Histomorphometrical calculation of relative osteoid area (O.Ar/B.Ar) in vertebral bodies L2 by Von Kossa/Van Gieson staining. (**f**) Von Kossa/Van Gieson staining in vertebral body L2 from an untreated mouse (red: osteoid; black: mineralized bone; brown: bone marrow). Mice were immunized with testicular homogenate in adjuvant (EAO), adjuvant alone (adjuvant) or remained untreated (control). ** *p* < 0.01; *** *p* < 0.001.

Osteoblast activity was determined by the expression of alkaline phosphatase (ALP), a major enzyme of osteoblast hydroxyapatite synthesis. Enzyme histochemical staining of ALP in vertebral bodies resulted in a distinctive violet labeling located along the trabecular surface (Figure 9a). Histomorphometrical calculation illustrated a significantly decreased relative ALP perimeter (ALP.Pm/Tb.Pm, Figure 9b) in the EAO (12.720 ± 1.841%) and adjuvant groups (21.298 ± 3.237%) compared to control (43.185 ± 4.818%, *p* < 0.001 vs. EAO, *p* < 0.01 vs. Adj). This finding was supported by a reduced mRNA expression of ALP (Figure 9c) in the immunized groups (EAO −9.034 ± 0.102 [−∆CP], Adj −9.017 ± 0.106 [−∆CP]) in contrast to control (−8.461 ± 0.110 [−∆CP], *p* < 0.01 each) determined by real-time reverse transcription polymerase chain reaction (real-time RT-PCR). Beside bone mineralization, osteoblasts are responsible for synthesis and secretion of organic bone matrix. Therefore, we examined the mRNA expression of collagen 1α1 (Col1α1, Figure 9d) as another parameter of osteoblast activity. Regarding the mRNA expression of collagen 1α1, we detected no significant differences between the investigated groups (EAO −2.092 ± 0.256 [−∆CP], Adj −2.490 ± 0.082 [−∆CP], Cont −2.411 ± 0.143 [−∆CP]). Additionally, we conducted Von Kossa/Van Gieson staining of histological specimens, which resulted in a red labeling of osteoid (Figure 9f). However, the histomorphometrical calculation of relative osteoid area (O.Ar/B.Ar, Figure 9e) influenced neither EAO nor adjuvant compared to control (EAO 21.674 ± 1.662%, Adj 17.720 ± 4.299%, Cont 15.695 ± 3.673%). Detailed results are shown in Table S6.

### 2.5. Osteoclasts

**Figure 10:**
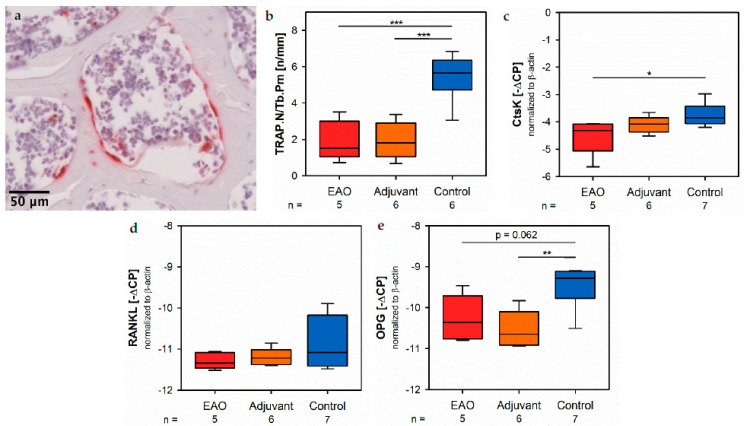
Osteoclasts. (**a**) Enzyme histochemical staining of tartrate resistant acid phosphatase (TRAP, red) in vertebral body L3 from an untreated mouse. (**b**) Histomorphometrical calculation of number of TRAP positive cells per trabecular perimeter (TRAP.N/Tb.Pm) in enzyme histochemical-stained vertebral bodies L3. mRNA expression of (**c**) cathepsin K (CtsK), (**d**) receptor activator of nuclear factor-kappa B ligand (RANKL) and (**e**) osteoprotegerin (OPG) in real-time RT-PCR of vertebral bodies Th10. Mice were immunized with testicular homogenate in adjuvant (EAO), adjuvant alone (adjuvant) or remained untreated (control). * *p* < 0.05; ** *p* < 0.01; *** *p* < 0.001.

Osteoclasts were identified by enzyme histochemical staining of tartrate resistant acid phosphatase (TRAP). Multinuclear, red stained cells in contact to the trabecular surface were counted as osteoclasts (Figure 10a). The calculation of osteoclast number in relation to trabecular perimeter (TRAP.N/Tb.Pm, Figure 10b) illustrated a significant reduction of osteoclasts in EAO (1.924 ± 0.488 n/mm) and adjuvant groups (1.933 ± 0.407 n/mm) compared to control (5.447 ± 0.527 n/mm, *p* < 0.001 each). These findings were supplemented by the results of real-time RT-PCR regarding cathepsin K (CtsK), another typical degrading enzyme of osteoclasts. The mRNA expression of cathepsin K (Figure 10c) was significantly decreased after EAO (−4.522 ± 0.292 [−∆CP]) in comparison to control (−3.754 ± 0.159 [−∆CP], *p* < 0.05) while it was not significantly decreased in the adjuvant group (−4.097 ± 0.129 [−∆CP], *p* = 0.620). As a major regulatory factor of osteoclast differentiation, we determined the mRNA expression of the receptor activator of nuclear factor-kappa B ligand (RANKL, Figure 10d) but no significant alterations were detected (EAO −11.292 ± 0.088 [−∆CP], Adj −11.192 ± 0.085 [−∆CP], Cont −10.816 ± 0.237 [−∆CP]). Additionally, we evaluated the mRNA expression of osteoprotegerin (OPG, Figure 10e), an inhibitor of RANKL and, thereby, regulating the differentiation of osteoclasts. Expression of osteoprotegerin was significantly reduced in the adjuvant group (−10.530 ± 0.186 [−∆CP], *p* < 0.01 vs. Cont −9.493 ± 0.194 [−∆CP]) and almost significantly decreased after EAO (−10.262 ± 0.249 [−∆CP], *p* = 0.062 vs. Cont). Therefore, the reduction of osteoclast activity can neither been explained by RANKL nor by osteoprotegerin. Detailed results are shown in Table S7.

### 2.6. Osteocytes and Cell-Contacts

**Figure 11:**
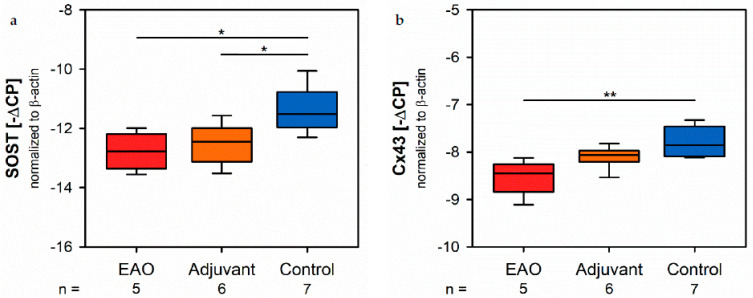
Osteocytes and cell-contacts. mRNA expression of (**a**) sclerostin (SOST) and (**b**) connexin 43 (Cx43) in real-time RT-PCR of vertebral bodies Th10. Mice were immunized with testicular homogenate in adjuvant (EAO), adjuvant alone (adjuvant) or remained untreated (control). * *p* < 0.05; ** *p* < 0.01.

Since sclerostin represents a typical marker of osteocytes, we analyzed the mRNA expression of the sclerostin gene (SOST, Figure 11a) by real-time RT-PCR. The mRNA expression of SOST was significantly decreased in both immunized groups (EAO −12.782 ± 0.277 [−∆CP], Adj −12.522 ± 0.281 [−∆CP]) compared to control (−11.359 ± 0.295 [−∆CP], *p* < 0.05 each). Gap junctions link osteocytes with each other and are mandatory regarding structural integrity and cellular survival. Connexin 43 represents a characteristic element of gap junctions. The mRNA expression of connexin 43 (Cx43, Figure 11b) was significantly lower after EAO (−8.530 ± 0.164 [−∆CP]) than it was in the control group (−7.759 ± 0.122 [−∆CP], *p* < 0.01) while the adjuvant group showed an intermediate mRNA level of connexin 43 (−8.100 ± 0.097 [−∆CP], *p* = 0.199 vs. Cont). Detailed results are shown in Table S8.

### 2.7. Biomechanical Properties

**Figure 12:**
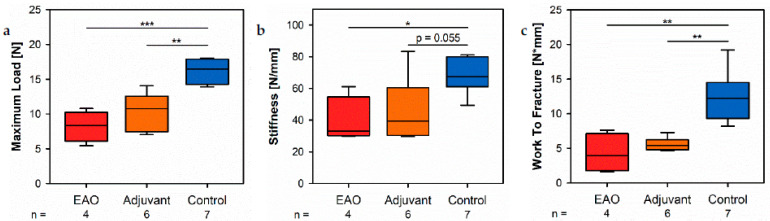
Biomechanical properties of the femora in the three-point bending test. Measurement of (**a**) maximum load, (**b**) stiffness and (**c**) work to fracture. Mice were immunized with testicular homogenate in adjuvant (EAO), adjuvant alone (adjuvant) or remained untreated (control). * *p* < 0.05; ** *p* < 0.01; *** *p* < 0.001.

By a three-point bending test of the femora, we objectified the consequences regarding biomechanical bone properties due to the cellular and molecular alterations following EAO described before. The maximum load (Figure 12a) was significantly decreased in the EAO (8.234 ± 1.099 N) and adjuvant groups (10.392 ± 1.086 N) compared to control (15.984 ± 0.665 N, *p* < 0.001 vs. EAO, *p* < 0.01 vs. Adj). The stiffness (Figure 12b) was significantly reduced in the EAO group (39.331 ± 7.355 N/mm, *p* < 0.05 vs. Cont 66.980 ± 4.208 N/mm) and almost significantly reduced in the adjuvant group (45.886 ± 8.284 N/mm, *p* = 0.055). Consequently, both immunized groups required less work to fracture compared to control (Figure 12c, EAO 4.277 ± 1.421 N/mm, Adj 5.566 ± 0.390 N/mm, Cont 12.520 ± 1.380 N/mm, *p* < 0.01 Cont vs. each). Solely, the post yield displacement (Figure S2) showed no significant alteration (EAO 0.982 ± 0.430 mm, Adj 0.746 ± 0.151 mm, Cont 0.968 ± 0.123 mm). Detailed results are shown in Table S9.

### 2.8. Body Weight

Mice were weighed before the first immunization and 50 days after the first immunization in order to check their general health and well-being. Regarding the initial body weight (Table 1, Body weight before) as well as the body weight before euthanasia (Table 1, Body weight after) no differences between the investigated groups were noticed. Furthermore, all groups significantly gained body weight during the period of 50 days.

## 3. Discussion

In the present study we investigated the bone tissue in a well-established mouse model of experimental autoimmune-orchitis (EAO) and compared it to an adjuvant control (adjuvant) and untreated control group (control). The advantage of using an animal model of testicular inflammation lies in direct traceability and reproducibility in contrast to human orchitis, which often is diagnosed late because of indirect symptoms like fertility disorder. Therefore, we assumed the mouse model of EAO would enable an unaffected investigation of side effects following epididymo-orchitis, such as influences on bone metabolism.

Histomorphometrical analysis of vertebrae stained with hematoxylin and eosin illustrated a reduced bone area, while Tb.Pm, Tb.Th and Tb.Sp remained unaltered following EAO. In contrast, µCT of trabecular regions in the femora and vertebrae demonstrated a reduction of Tb.N and Tb.Th with an increase of Tb.Sp resulting in a lowered BV/TV and BS/TV due to EAO and adjuvants, which are typical characteristics of osteoporotic bones. The increase of BS/BV pronounced the loss of trabecular bone volume over trabecular bone surface. The benefit of investigations by µCT is based on the evaluation of a defined bone volume of interest containing several slices at the same time, while histomorphological analysis often includes only a single slice per specimen because of material shortage and the required time. Therefore, µCT offers a more comprehensive and reliable assessment of bone tissue than histomorphometry. Furthermore, µCT enables an investigation of the three-dimensional orientation and connectedness of the trabecular network, commonly illustrated by the Tb.Pf and SMI [15,16]. After EAO and immunization with adjuvants alone, the Tb.Pf as well as the SMI were elevated and, thereby, indicated a loss of intertrabecular connectedness, bone mass and structural stability. µCT of the femoral cortex at mid and distal diaphysis demonstrated that the Ct.Th was significantly decreased due to EAO and adjuvants, which was confirmed by an increase of Ct.S/Ct.V, indicating a loss of cortical bone volume because the Ct.S remained nearly unaltered. The cortical porosity was not determined because of the small pore size and limited resolution of our µCT analysis with a 5.75 µm isotropic voxel side length. BMD and TMD were decreased at all sites analyzed by µCT. Thus, EAO and adjuvants induced not only a reduction of bone mass and trabecular connectedness but also a deficiency in bone mineralization, thereby weakening the bone strength and fulfilling the criteria of osteoporosis. In summary, the alterations in µCT following EAO and immunization with adjuvants alone were similar to the findings in a mouse model of senile osteoporosis [17].

The biomechanical investigation of the femora by a three-point bending test demonstrated a decreased maximum load and stiffness due to EAO and adjuvants. Both parameters depend on bone morphology and bone material properties [18]. Therefore, the three-point bending test confirmed the findings of the structural evaluation by µCT and the expected reduction of bone stability. The post yield displacement remained without major changes, thus indicating no significant changes regarding the bone matrix composition and organization [18]. Depending on all three previously mentioned parameters, the work to fracture illustrated a decreased overall resistance of the femora to failure after EAO and immunization with adjuvants [18]. In summary, the three-point bending test visualized the consequences of a reduced bone mass and mineralization. Diminished bone strength is a commonly found characteristic of osteoporotic bones and is accompanied by a growing risk of osteoporotic fractures [19].

The present study illustrated that osteoporosis caused by EAO and adjuvants can be explained by altered bone metabolism. The mRNA expression of ALP, as well as the enzyme histochemical staining of ALP, indicated a lowered synthesis and release of ALP by osteoblasts due to EAO and adjuvants. Thereby, we identified a decreased osteoblast activity after EAO as well as immunization with adjuvants resulting in less hydroxyapatite synthesis and bone mineralization, as we demonstrated by TMD and BMD in the µCT investigation. In contrast, the mRNA expression of collagen 1α1 remained unaltered in the EAO and adjuvant groups. Type I collagen represents the most abundant type of collagen in bone tissue and is mainly synthesized by osteoblasts [20]. Therefore, type I collagen is a crucial part of the bone matrix composition and its unaltered expression provides an explanation for the unaffected post yield displacement in the three-point bending test. Additionally, the Von Kossa/Van Gieson staining of histological sections and subsequent calculation of the relative osteoid area (O.Ar/B.Ar) demonstrated an unaffected osteoid formation by osteoblasts after immunization. However, alterations regarding collagen fiber arrangement, regulation of mRNA and protein level, as well as collagen products in blood serum, are common findings in animal models of osteoporosis and human osteoporosis patients [21,22,23]. The differences between the decreased mineralization activity in contrast to the remaining collagen and osteoid synthesis may suggest a differentiated regulation of osteoblast activity and specific enzymes in the case of EAO and immunization with adjuvants alone, different from other animal models and human osteoporosis in the elderly.

Usually, reduced bone mass is caused by an increased number and/or enzymatic activity of osteoclasts. However, in the present study we illustrated a decline of CtsK by real-time RT-PCR and osteoclast number by enzyme histochemical staining of TRAP, both characteristic osteoclastic enzymes responsible for degradation of organic bone matrix. Thus, we determined the mRNA expression of RANKL, representing a major signal molecule that induces osteoclast differentiation and is synthesized by osteoblasts. However, there was no significant alteration to be reported after immunization [24]. In addition, we analyzed the mRNA expression of OPG, another regulator protein of osteoclast differentiation that inhibits the binding of RANKL to its receptor and is synthesized by osteoblasts as well [25]. In the adjuvant group we noticed a significant decrease, and in the EAO group a nearly significant decrease, of OPG. In combination with the unaffected expression of RANKL a reduction of OPG would be expected to cause increased osteoclastic activity and indicates the efforts of osteoblasts to stimulate the turnover of bone tissue. However, on the contrary, we found a decline in osteoclastic activity after immunization, as described above. This may illustrate the greater relevance of further mechanisms that regulate the differentiation and activity of osteoclasts in the case of the applied immunizations and the need for more detailed investigations. However, whole bone metabolism by osteoblasts and osteoclasts seems to be downregulated following EAO, as well as by immunization with adjuvants alone. However, the osteoblast activity must be significantly more inhibited than the osteoclast activity to result in a reduced bone mass overall as we showed by histomorphometrical evaluation and µCT. This indicates similarities with senile osteoporosis, where general turnover of bone tissue becomes progressively more inactive as well [26].

Osteocytes detect the mechanical stress of bone tissue and regulate osteogenesis by osteoblasts, as well as bone resorption by osteoclasts, and consequently have a crucial role regarding fracture healing and remodeling of bone tissue [27]. One of the most important regulatory proteins synthesized solely by osteocytes is sclerostin, which is encoded by the SOST gene and acts as a link between mechanical stimuli and osteoblastic bone formation [27]. An upregulation of SOST is known to inhibit bone formation by osteoblasts [27]. The present investigation determined a decreased mRNA expression of SOST following immunization, which should benefit increased osteoblastic bone formation. However, the synthesis and release of ALP, as well as bone mineralization, were reduced in consequence of EAO and immunization with adjuvants, while the expression of collagen 1α1 and osteoid formation remained on a similar level compared to the control group. Therefore, we suggest that sclerostin more likely affects collagen synthesis and osteoid formation than bone mineralization by osteoblasts in the context of EAO and after application of adjuvants, but the specific underlying mechanisms must be further investigated. However, sclerostin does not provide a sufficient explanation for the observed reduction of bone mass and mineralization in this study.

Cx43 represents the most abundant connexin in bone tissue and is found in osteocytes, osteoblasts and osteoclasts [28]. It is a transmembrane protein and an essential element of hexametric hemichannels, called connexons [28]. These combine with connexons of neighboring cells and build gap junctions [28]. In this way, Cx43 essentially contributes to osteoblast differentiation, cellular survival, maintenance of bone area fraction, bone mineralization, bone material strength and fracture healing [29]. Therefore, the detected reduction of Cx43 mRNA expression caused by EAO indicates a significant remodeling of the intercellular network leading to a loss of cell-cell-contacts and may provide an explanation for the decreased osteoblast activity, bone mineralization, bone mass and biomechanical properties due to EAO.

Summarizing, after induction of EAO we detected several alterations regarding bone status, which indicated the development of osteoporosis in the utilized mouse model. However, the majority of parameters were also significantly altered in the same direction after immunization with adjuvants, but without testicular homogenate. Therefore, the main finding of the present study is that adjuvants alone cause significant bone effects in the mouse model and probably are responsible for the majority of effects in the EAO group within the investigated period of 50 days, thereby obscuring specific effects of the autoimmune-orchitis itself. Nevertheless, some parameters demonstrated significantly stronger effects in the EAO group compared to the adjuvant group, such as the µCT of the trabecular region in vertebrae L1. Other parameters indicated significant alterations in the EAO group compared to control, but not in the adjuvant group compared to control, such as cortical BMD and TMD, CtsK and Cx43. Therefore, EAO at least was able to enhance a few bone effects compared to adjuvants alone within 50 days. Due to the slow metabolism of bone tissue, a longer investigation period may improve the bone effects of EAO in contrast to adjuvants alone in future studies. However, at the moment, and based on the present data, the investigated mouse model of EAO with a test period of 50 days cannot be recommended for research purposes to investigate the bone effects of orchitis, because adjuvants represent a major confounder and, therefore, the transfer of laboratory findings into a clinical application is limited.

We suggest that these bone effects are mainly caused by the concomitant hypogonadism related to the lack of systemically available testosterone. However, we did not evaluate the serum levels of any hormones in the mouse model of EAO, but considering the significant results of the present study relevant hormones must be determined in future studies. Nevertheless, this assumption is based on the evidence of decreased testosterone serum levels in the EAO and adjuvant groups in a rat model of EAO [6] as well as the importance of androgens representing crucial regulators of bone metabolism in men [10,11,12]. In contrast, several experimental models of EAO demonstrated an increased intratesticular testosterone production of Leydig cells [6,30,31,32]. This discrepancy between elevated intratesticular testosterone production and a decline in testosterone serum level could be explained by alterations regarding blood flow, or a reduction in capillary permeability in damaged testicles, leading to a decreased release of testosterone from the testicles, or by alterations in the metabolic clearance of testosterone [32]. Beside diminished testosterone serum levels, direct effects of the immunization regarding bone metabolism must also be considered. To our knowledge no other organs beside testicles and epididymides have been investigated in rodent models of EAO so far. Therefore, we cannot estimate whether the autoimmune effects following the application of testicular homogenate are actually organ-specific to testicles and epididymides, as has been described before, or whether other organs and tissues, like bones for example, are directly affected by the autoimmune reaction as well [33]. On the other hand previous investigations illustrated that the supplemental application of complete Freund’s adjuvant (CFA) and Bordetella pertussis toxin, beside testicular homogenate, is mandatory to evoke severe EAO [34]. However, treatment with the adjuvants alone can also evoke autoimmune reactions against some testicular autoantigens despite the use of no testicular homogenate [34]. Considering the significant bone effects solely of the adjuvants in the present study raises the question whether the adjuvants may cause autoimmune effects in the bone tissue too. This suggestion is supported by the already established use of CFA to induce polyarthritis in a rodent model [35]. However, specific investigations regarding bone status after administration of CFA and Bordetella pertussis toxin have not been conducted so far. However, we did not observe any obvious inflammatory infiltrates in the histological specimens of vertebrae in the present study, but additional inflammatory parameters of bone tissue might be investigated by real-time RT-PCR in future studies. Furthermore, it has been shown in vitro and in vivo, that Mycobacterium tuberculosis is able to inhibit osteoclastogenesis by the production of the chaperonin protein Cpn60.1 [36]. On the contrary, the Mycobacterium tuberculosis in CFA was inactivated before immunization and histomorphological evaluation did not reveal any accumulation of bacteria in the investigated bone tissue. In vitro and in vivo Bordetella pertussis toxin itself was even found to be beneficial regarding bone metabolism by blocking endogenous G protein–coupled receptor-driven G_i_-signaling in osteoblasts, and induced an increase in bone volume in aging female mice [37,38]. Summarizing, the actual reason for the significant alterations regarding bone status in the adjuvant group cannot finally be explained by the present study and needs to be elucidated by a future study.

There are some further impact factors that may especially contribute to the slightly enhanced alterations of bone metabolism after EAO. Beside effects regarding testicles themselves, previous investigations of EAO revealed disturbances of the hypothalamic-testicular axis. In rat models of EAO, increased serum levels of luteinizing hormone (LH) and follicle-stimulating hormone (FSH) were detected [6,30,31,32]. These gonadotropins influence the activity of osteoblasts and osteoclasts as well. While LH supports bone formation by osteoblasts, increases of FSH are associated with a reduction in BMD [39,40]. Considering the greater increase of FSH compared to LH in previous studies, alterations of gonadotropins may contribute to the reduction of bone mass and mineralization in EAO models [6,32]. Osteocalcin represents another messenger molecule that illustrates the interactions between bone tissue and testicles [41]. It is synthesized by osteoblasts, excreted in the extracellular bone matrix and regulates bone formation [42]. Following a decarboxylation process, the undercarboxylated osteocalcin is released into the systemic blood stream and exerts a variety of regulatory functions [41,43]. Undercarboxylated osteocalcin stimulates testosterone biosynthesis by Leydig cells and favors male fertility [44]. In the context of orchitis, the impaired testicular release of testosterone is assumed to initially decrease bone metabolism as the present study demonstrated. Due to a decreased osteoblast activity, we would expect a decline of undercarboxylated osteocalcin serum level following EAO. This would result in a suppression of testosterone production and enhanced fertility disorder. Summarizing, the interaction between bone tissue and testicles by androgens and osteocalcin may result in a vicious circle that could continue even when the initial trigger, such as orchitis, subsided. Therefore, further investigations are required in order to elucidate the role of osteocalcin in the context of orchitis. On the contrary, primary diseases of bone tissue like multiple myeloma, which cause a decline in osteoblast activity, as well as systematic diseases like Crohn’s disease, may lead to lowered osteocalcin serum levels and thereby provoke male fertility disorder and the vicious circle described above [45,46].

In conclusion, the present study offers the first insight regarding the consequences of EAO on bone tissue. We illustrated significant alterations regarding bone metabolism, bone mass, bone microarchitecture and bone mineralization resulting in decreased biomechanical stability. These alterations may partly be explained by decreased serum testosterone levels, but further investigations are required to elucidate the relevance of additional impact factors such as LH and FSH. Moreover, the role of the hypothesized feedback mechanism between testicles and bone tissue by osteocalcin and androgens in the case of an initial hypogonadism has to be further evaluated. However, significant alterations following immunization with the adjuvants alone illustrate them to be responsible for the majority of bone effects. Therefore, the investigated mouse model of EAO with a test period of 50 days currently appears to be inappropriate to examine the effects of orchitis regarding bone metabolism.

## 4. Materials and Methods

### 4.1. Mouse Model of Experimental Autoimmune-Orchitis (EAO)

Rodent models of EAO are well established in the Department of Anatomy and Cell Biology at the Justus-Liebig-University Giessen [6,47]. For the present study 10–12 weeks old male C57BL/6 mice (Charles River Laboratories, Sulzfeld, Germany) were housed in the animal facility of the Justus-Liebig-University Giessen under specific pathogen free conditions with a 12 h day and night cycle at 20–22 °C and free access to water and food. EAO was induced by active immunization with testicular homogenate in complete Freund’s adjuvant (CFA; Sigma-Aldrich Inc., Saint Louis, MO, USA) as previously described [47]. Testicular homogenate was prepared from decapsulated testes collected from adult syngeneic mice and homogenized in sterile 0.9% NaCl at a ratio of 1:1. For immunization, testicular homogenate was mixed with CFA at a ratio of 1:1. Per immunization four subcutaneous injections with a total volume of 200 µL (50 µL per injection site) were conducted dorsally under anesthesia by inhalation of 3–5% isoflurane (EAO, *n* = 5). Simultaneously intraperitoneal injection of 100 ng Bordetella pertussis toxin (Calbiochem, Merck KGaA, Darmstadt, Germany) in 100 µL Munõz Buffer (25 mM Tris, 0.5 M NaCl, 0.017% Triton X-100, pH 7.6) [48] was carried out in order to boost the immune reaction. In total, animals were immunized three times every 14 days. Periprocedural analgesia was provided in form of Tramadol (STADApharm GmbH, Bad Vilbel, Germany) in drinking water (2.5 mg/mL) started 24 h before each immunization and continued for the following three days. As an adjuvant control group, age-matched mice received CFA mixed with 0.9% NaCl instead of testicular homogenate and Bordetella pertussis toxin following the same procedure (adjuvant, *n* = 6). An additional control group of age-matched mice remained completely untreated (control, *n* = 7). Fifty days after the first immunization, mice were deeply anesthetized by inhalation of 5% isoflurane and euthanized by cervical dislocation. Immediately after certain death, several bones were explanted, carefully cleaned from excessive tissue and preserved for the intended investigations. Vertebrae Th10 (real-time RT-PCR) were incubated in RNAlater stabilization solution (Thermo Fisher Scientific Inc., Waltham, MA, USA) as quickly as possible and stored at −80 °C. Vertebrae L1 (µCT), L2 (embedding in Technovit 9100 for histology), L3 (embedding in paraffin for histology) and right femora (µCT) were fixed in 4% phosphate buffered paraformaldehyde at 4 °C. Left femora (three-point bending test) were enveloped in gauze bandage, moistened with 0.9% NaCl and stored at −20 °C. Especially bones from the EAO group appeared more fragile during explantation and preparation. If a bone fractured during the explantation and preparation process, it was excluded from the subsequent evaluation.

Execution of animal experiments was authorized by the responsible licensing body of regional ethical committee on animal care (regional council Giessen, Germany, reference number “AZ 58/2014”). All animal experiments were carried out in accordance with the EU Directive 2010/63/EU [49] and the guidelines for the care and use of laboratory animals of the German Animal Welfare Act. All methods were carried out by following the approved guidelines.

### 4.2. Hematoxylin-Eosin Staining (HE)

For various histological investigations, vertebrae L3 were fixed in 4% phosphate buffered paraformaldehyde at 4–8 °C for 24 h. Subsequently, they were washed in 0.1 M phosphate buffer (pH 7.3) and decalcified in 10% ethylenediaminetetraacetate (EDTA) until a rubbery consistency was achieved. After the EDTA was washed out in water vertebrae were dehydrated with an ascending series of ethanol followed by xylol and finally embedded in paraffin. Thin sections (5 µm) of vertebrae L3 were cut in horizontal orientation using a rotary microtome (Microm HM 355S, Thermo Fisher Scientific Microm International GmbH, Walldorf, Germany). Sections were deparaffinized in xylol, rehydrated and stained with Mayer’s hemalaun and eosin. Finally, they were dehydrated with an ascending series of ethanol followed by xylol and cover slipped with DePex mounting medium (VWR International GmbH, Darmstadt, Germany).

For histomorphometrical evaluation, overlapping microscopic images of the whole vertebral bodies were taken at 40× magnification (microscope: DM5500 B, camera: DFC7000 T, Leica Microsystems CMS GmbH, Wetzlar, Germany). Then, single images were merged to receive an overview image of the complete vertebral body (LAS X, version 3.7.3.23245, Leica Microsystems CMS GmbH, Wetzlar, Germany). Histomorphometrical analysis itself was performed using Fiji Image J (version 2.1.0/1.53c, National Institutes of Health, Bethesda, MD, USA) [50]. In HE-stained sections, the total bone area (B.Ar) of vertebral bodies was measured by the Trainable Weka Segmentation plugin [51]. Trabecular thickness (Tb.Th) and trabecular separation (Tb.Sp) were determined by BoneJ plugin [52]. The trabecular perimeter (Tb.Pm) was measured manually in Fiji ImageJ.

### 4.3. Enzyme Histochemical Staining of Alkaline Phosphatase (ALP)

Deparaffinized and rehydrated 5 μm thin sections of decalcified vertebrae L3 were used for enzyme histochemical staining of ALP as described previously [53]. Sections were kept in 0.1 M Tris buffer (pH 9.4) for 10 min. Subsequently, ALP detection was achieved by incubation with phosphatase substrate solution consisting of 5-bromo-4-chloro-3-indolylphosphate and nitroblue tetrazolium (BCIP/NBT; KPL, Gaithersburg, MD, USA) in a humidity chamber at 37 °C for 30 min. After washing with distilled water, counterstaining was conducted with nuclear fast red-aluminum sulfate solution (Carl Roth GmbH + Co. KG, Karlsruhe, Germany) for 10 min. Excessive stain was washed off with distilled water. Sections were dehydrated with an ascending series of ethanol followed by xylol and cover-slipped with Eukitt mounting medium (Sigma-Aldrich Chemie GmbH., Taufkirchen, Germany).

For histomorphometrical evaluation, overlapping microscopic images of the whole vertebral bodies were taken at 10× magnification and merged to create overview images as described above. In these overview images the ALP positive part of the trabecular perimeter (violet) was calculated in relation to the total trabecular perimeter (relative ALP perimeter, ALP.Pm/Tb.Pm) with Fiji ImageJ.

### 4.4. Enzyme Histochemical Staining of Tartrate Resistant Acid Phosphatase (TRAP)

Thin sections (5 μm)of decalcified vertebrae L3 were deparaffinized in xylol and rehydrated in order to use them for enzyme histochemical staining of TRAP, as described previously [54]. Briefly, sections were preincubated in 0.1 M sodium acetate buffer (pH 5.2) for 10 min. TRAP detection was accomplished by incubation with a sodium acetate buffered solution of naphthol AS-TR phosphate (Sigma-Aldrich Chemie GmbH, Taufkirchen, Germany), N,N-dimethylformamide (Sigma-Aldrich), di-sodium tartrate dihydrate (Merck KGaA, Darmstadt, Germany) and fast red TR salt (Sigma-Aldrich) in a humidity chamber at 37 °C for 30 min. Sections were counterstained with Shandon instant-hematoxylin (Thermo Scientific, Schwerte, Germany) and cover-slipped with Kaiser’s glycerin gelatin (Carl Roth GmbH + Co. KG, Karlsruhe, Germany).

Regarding histomorphometrical analysis, multinuclear, red-labeled cells with contact to the trabecular surface were classified as osteoclasts. Vertebral bodies were systematically investigated at 40× magnification. Only images that contained osteoclasts were taken. Overlapping of images was avoided in order to prevent multiple counting of osteoclasts. Finally, the total number of osteoclasts in all images of an individual specimen was determined and calculated in relation to the trabecular perimeter (TRAP.N/Tb.Pm) by using Fiji ImageJ.

### 4.5. Von Kossa/Van Gieson Staining

Vertebrae L2 were also fixed in 4% phosphate buffered paraformaldehyde and washed in 0.1 M phosphate buffer (pH 7.3). By contrast, vertebrae L2 were not decalcified before they were dehydrated with an ascending series of ethanol and embedded in Technovit 9100 (Kulzer GmbH, Hanau, Germany) as described in detail by the manufacturer’s protocol. Thin (5 µm) sections of vertebrae L2 were cut in horizontal orientation using a rotary microtome (RM2155, Leica Microsystems GmbH, Wetzlar, Germany). These sections were stained with the Von Kossa/Van Gieson method as described earlier [53]. Embedding medium was removed by incubating sections with 2-methoxyethyl acetate (Merck KGaA, Darmstadt, Germany). After rehydration and incubation in 3% silver nitrate solution (Sigma-Aldrich Chemie GmbH, Taufkirchen, Germany), sections were washed in distilled water and incubated in 10% sodium carbonate formaldehyde solution (Merck). Slides were rinsed under running tap water before incubation in 5% sodium thiosulfate solution (Merck). After washing in distilled water counterstaining was conducted with methyl green (Carl Roth GmbH + Co. KG, Karlsruhe, Germany). Again, slides were rinsed under running tap water and distilled water before they were incubated in Weigert’s iron hematoxylin solution (Roth) for nuclear staining. After washing in tap water, slides were incubated in Van Gieson’s mixture (Waldeck GmbH & Co. KG, Münster, Germany). Finally, sections were dehydrated in ethanol and xylol before cover-slipping with DePex mounting medium (VWR International GmbH, Darmstadt, Germany).

For histomorphometrical evaluation, overlapping microscopic images of the whole vertebral bodies were taken at 10× magnification and merged to create overview images as described above. After Von Kossa/Van Gieson staining mineralized bone tissue appeared black, osteoid was red and bone marrow had a brown color. The osteoid area was measured in relation to the total bone area (O.Ar/B.Ar) by the Trainable Weka Segmentation plugin of Fiji ImageJ [50,51].

### 4.6. Real-Time Reverse Transcription Polymerase Chain Reaction (Real-Time RT-PCR)

Vertebrae Th10 were transferred into RNAlater stabilization solution (Thermo Fisher Scientific Inc., Waltham, MA, USA) immediately after explantation. For isolation of RNA, specimens were homogenized in 1 mL QIAzol lysis reagent (Qiagen GmbH, Hilden, Germany) per 100 mg specimen by using a vibration mill (MM400, Retsch GmbH, Haan, Germany). Chloroform (200 µL) (Sigma-Aldrich Chemie GmbH, Taufkirchen, Germany) per 1 mL QIAzol were added before centrifugation with 14,000 rpm at 4 °C for 15 min. The supernatant containing the RNA was collected, mixed with 1.5× volume of 100% ethanol and transferred into RNeasy Mini Spin Columns (Qiagen). During centrifugation RNA bound to the column membrane. RNA was washed with several buffers (miRNeasy Mini Kit, Qiagen) following the manufacturer’s protocol. Finally, RNA was eluted with RNase-free water. Concentration and purity of RNA were determined by a spectrophotometer (ND-1000, NanoDrop Technologies LLC., Wilmington, DE, USA). Isolated RNA was stored at −80 °C until reverse transcription into cDNA.

Isolated RNA (750 ng) was incubated with 3 µL gDNA wipeout buffer (Qiagen) at 42 °C in a thermo cycler (TC-3000, Techne Inc., Burlington, NJ, USA; Bibby Scientific Ltd., Staffordshire, UK) in order to eliminate contaminations with genomic DNA. Afterwards 250 ng RNA were separated for negative control. Quantiscript reverse transcriptase (RT), Quantiscript RT buffer and RT primer mix (QuantiTect Reverse Transcription Kit, Qiagen) were added according to the manufacturer’s protocol. cDNA was synthesized during incubation for 30 min at 42 °C. Then, reverse transcriptase was inactivated at 95 °C for 3 min. Samples were stored at −20 °C until further processing.

Real-time RT-PCR was performed using the Quantifast SYBR Green PCR Kit (Qiagen) according to the manufacturer’s protocol. Primers (0.1 µL) (Table S10, MWG-Biotech AG, Ebersberg, Germany), 3.9 µL RNase-free water and 5 µL Quantifast Mastermix (Qiagen) were mixed with 1 µL cDNA or RNase-free water to a total volume of 10 µL. The dilution was transferred into capillaries (Roche Molecular Systems Inc., Basel, Switzerland), centrifuged at 2000 rpm for 20 s and incubated in a LightCycler 2.0 (Roche) with the following protocol: initial denaturation at 95 °C for 5 min, then 40 cycles consisting of 10 sec denaturation at 95 °C and 30 s annealing and elongation at 60 °C. Afterwards, the purity of PCR product was analyzed by melting curve analysis with the LightCycler software (version 4.1, Roche). During real-time RT-PCR, the following controls were executed: (a) cDNA synthesis without addition of reverse transcriptase in order to check for contaminations with genomic DNA, (b) PCR without template, (c) evaluation of PCR efficiency and slope and (d) samples were analyzed in double determination. The crossing point of the amplification curve and the horizontal threshold determined the CP-value, which was normalized to the reference gene β-actin in each sample.

### 4.7. Micro Computed Tomography (µCT)

Right femora and vertebrae L1 were fixed in 4% phosphate buffered paraformaldehyde, washed in 0.1 M phosphate buffer (pH 7.3) and scanned with a high-resolution micro computed tomograph (SkyScan 1173, Bruker microCT, Kontich, Belgium). The X-ray source voltage was set to 40 kV and source current to 200 µA. Scanning was performed by rotating samples 180° around the vertical axis in 0.24° rotation steps. Raw data images were captured with an exposure time of 1600 ms and a four-fold frame averaging for noise reduction. Reconstruction of cross-sectional images was conducted with NRecon Reconstruction Software (version 1.6.10.2, Bruker microCT). The resulting resolution was 5.75 μm isotropic voxel side length with an 8-bit gray-scale resolution. The cross-sectional data sets were adjusted using Data-Viewer (version 1.5.2.5, Bruker microCT). Subsequent analysis of micro computed tomography scans was executed with CT-analyzer software (version 1.16.4.1, Bruker microCT). A distal femoral growth plate was utilized as a reference level to define the volume of interest (VOI). At a distance of 0.216 mm from reference level, the trabecular area of distal femoral metaphysis was measured involving 309 slices (1.753 mm). The femoral cortex was analyzed at the distal diaphysis and mid diaphysis located 2.179 mm and 5.497 mm from reference level, each including 80 slices (0.45 mm). In accordance with the manual “Structural parameters measured by the Skyscan^TM^ CT-analyzer software” and the review of Bouxsein et al. [55], regions of interest (ROI) were selected with a short distance to the endocortical surface. In vertebral bodies, the trabecular area was manually selected excluding primary spongiosa and cartilage. Calibrating phantoms with characteristic calcium hydroxyapatite densities of 250 mg/cm^3^ and 750 mg/cm^3^ were used to calculate bone mineral density (BMD) and tissue mineral density (TMD).

Regarding the investigated areas of femoral cortex, the following parameters were evaluated: cortical thickness (Ct.Th), cortical surface (Ct.S), cortical surface/volume ratio (Ct.S/Ct.V), bone mineral density (BMD) and tissue mineral density (TMD). The following parameters were evaluated for the trabecular areas of the femora and vertebral bodies L1: bone volume fraction (BV/TV), bone surface density (BS/TV), specific bone surface (BS/BV), trabecular number (Tb.N), trabecular thickness (Tb.Th), trabecular separation (Tb.Sp), structure model index (SMI), trabecular pattern factor (Tb.Pf), bone mineral density (BMD) and tissue mineral density (TMD).

### 4.8. Biomechanical Analysis by Three-Point Bending Test

After explantation, the left femora were enveloped in a gauze bandage, moistened with 0.9% NaCl and stored at −20 °C. Before biomechanical testing, bones were slowly thawed. Femoral bones were centrally placed and perpendicularly aligned on a construction with 8.5 mm span length between both contact points (52.8% of average bone length). The force transducer (Xforce P, 500 N, ZwickRoell GmbH & Co. KG, Ulm, Germany) of the material testing machine (Z5.0 TN, ZwickiLine, ZwickRoell GmbH & Co. KG, Ulm, Germany) pushed down vertically on the femoral diaphysis in the middle between both supporting contact points in a single loading procedure until complete fracture was accomplished. Data were recorded by testXpert III software (version 1.11, ZwickRoell GmbH & Co. KG, Ulm, Germany) and displayed as a load-displacement curve with load on the *y*-axis and displacement on the *x*-axis. Subsequently, characteristic whole-bone biomechanical properties (maximum load, stiffness, post yield displacement, work to fracture) were calculated as described previously [18].

### 4.9. Statistical Analysis

Statistical analysis was executed using SPSS Statistics (version 23.0, IBM Corp., Armonk, NY, USA). After confirmation of normal distribution by Kolmogorov-Smirnov test a one-way analysis of variance (ANOVA) was conducted. Depending on the equality of variances, assessed by Levene’s test, a Bonferroni or Dunnett-T3 post hoc test followed the ANOVA. If normal distribution was rejected by the Kolmogorov-Smirnov test, the Mann-Whitney U test was applied followed by Bonferroni-Holm correction. The significance level was set at *p* < 0.05 (* *p* < 0.05, ** *p* < 0.01, *** *p* < 0.001). Results are reported as mean ± standard error of mean (SEM). Box-and-whisker plots were created with Sigma Plot (version 12.5, Systat Software Inc., San Jose, CA, USA).

## Figures and Tables

**Table 1:** Body weights before the first immunization and 50 days after the first immunization.

						*p*-Values		
Parameter	Statistic	Unit	EAO (*n* = 5)	Adjuvant (*n* = 6)	Control (*n* = 7)	EAO vs. Adj.	EAO vs. Cont.	Adj. vs. Cont.
Body weight before	$\bar{x}$ ± SEM	g	28.820 ± 1.233	27.283 ± 1.164	28.440 ± 0.680	0.978	1.000	1.000
Body weight after	$\bar{x}$ ± SEM	g	30.420 ± 1.283	29.783 ± 1.262	31.540 ± 0.963	1.000	1.000	0.938
*p*-values	before vs. after		0.020	0.001	0.016			

## Data Availability

Data are contained within the article and its Supplementary Materials.

## Supplementary Materials

The following are available online at https://www.mdpi.com/article/10.3390/ijms22157858/s1.
