# Supplementary material for: Bone Status in a Mouse Model of Experimental Autoimmune-Orchitis
Source: Int J Mol Sci. 2021 Jul 23;22(15):7858. doi: 10.3390/ijms22157858 (PMC8346031; doi:10.3390/ijms22157858)
Supplement: Supplementary file 1 [file ijms-22-07858-s001.zip › ijms-1281239-supplementary.pdf]

Bone Status in a Mouse Model of Experimental Autoimmune-Orchitis

Fabian Hemm, Monika Fijak, Jan Belikan, Marian Kampschulte, Thaqif El Khassawna, Adrian Pilatz, Christian Heiss, Katrin S. Lips

Supplementary Materials

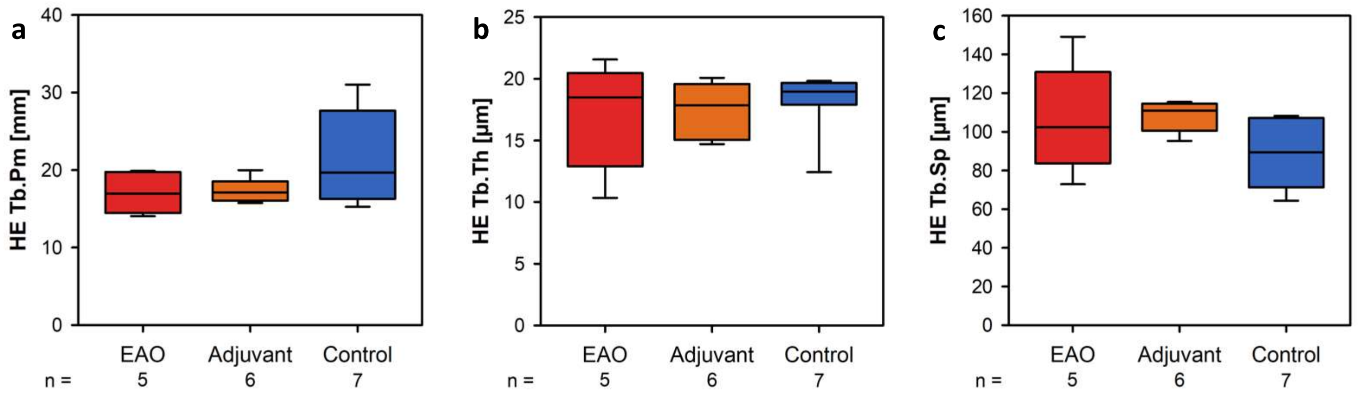

Figure S1. Histomorphometrical results of vertebral bodies L3 stained with hematoxylin-eosin (HE). Measurement of (a) trabecular perimeter (Tb.Pm), (b) trabecular thickness (Tb.Th) and (c) trabecular separation (Tb.Sp). Mice were immunized with testicular homogenate in adjuvant (EAO), adjuvant alone (adjuvant) or remained untreated (control).

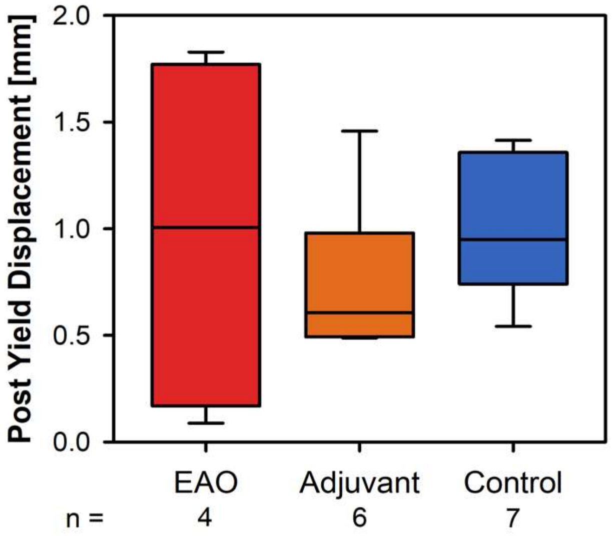

Figure S2. Post yield displacement of femora in the three-point bending test. Mice were immunized with testicular homogenate in adjuvant (EAO), adjuvant alone (adjuvant) or remained untreated (control).

Table S1. Detailed results of histomorphometrical analysis of vertebral bodies L3 stained with hematoxylin-eosin (HE). Measurement of bone area (B.Ar), trabecular perimeter (Tb.Pm), trabecular thickness (Tb.Th) and trabecular separation (Tb.Sp). Mice were immunized with testicular homogenate in adjuvant (EAO), adjuvant alone (adjuvant) or remained untreated (control). Data represent mean ($\bar{x}$) ± standard error of mean (SEM), lower quartile (Q_1), median quartile (Q_2), upper quartile (Q_3) and interquartile range (IQR).

Parameter	Statistic	Unit	EAO (n = 5)	Adjuvant (n = 6)	Control (n = 7)	p-values EAO vs. Adj.	EAO vs. Cont.	Adj. vs. Cont.
B.Ar	$\bar{x}$ ± SEM	mm^2	0.361 ± 0.020	0.453 ± 0.043	0.594 ± 0.048	0.491	0.005	0.080
	Q_1	mm^2	0.326	0.352	0.539			
	Q_2 (IQR)	mm^2	0.347 (0.078)	0.460 (0.203)	0.600 (0.081)			
	Q_3	mm^2	0.404	0.555	0.620			
Tb.Pm	$\bar{x}$ ± SEM	mm	17.082 ± 1.190	17.358 ± 0.658	21.550 ± 2.274	0.715	0.167	0.253
	Q_1	mm	14.469	16.053	16.275			
	Q_2 (IQR)	mm	16.963 (5.288)	17.087 (2.500)	19.689 (11.394)			
	Q_3	mm	19.757	18.553	27.669			
Tb.Th	$\bar{x}$ ± SEM	µm	17.050 ± 1.942	17.509 ± 0.904	18.024 ± 0.971	0.855	0.808	0.668
	Q_1	µm	16.225	14.929	17.894			
	Q_2 (IQR)	µm	18.934 (4.798)	18.405 (4.821)	18.971 (1.776)			
	Q_3	µm	21.023	19.750	19.670			
Tb.Sp	$\bar{x}$ ± SEM	µm	106.298 ± 12.552	108.196 ± 3.266	87.265 ± 6.338	1.000	0.176	0.109
	Q_1	µm	80.243	98.797	71.252			
	Q_2 (IQR)	µm	107.528 (59.804)	108.247 (15.716)	89.435 (35.950)			
	Q_3	µm	140.046	114.513	107.202			

Table S2. Detailed results regarding µCT investigation of mid diaphyseal femur cortex. Measurement of cortical thickness (Ct.Th), cortical surface (Ct.S), cortical surface/volume ratio (Ct.S/Ct.V), bone mineral density (BMD) and tissue mineral density (TMD). Mice were immunized with testicular homogenate in adjuvant (EAO), adjuvant alone (adjuvant) or remained untreated (control). Data represent mean ($\bar{x}$) ± standard error of mean (SEM), lower quartile (Q_1), median quartile (Q_2), upper quartile (Q_3) and interquartile range (IQR).

Parameter	Statistic	Unit	EAO (n = 5)	Adjuvant (n = 6)	Control (n = 7)	p-values EAO vs. Adj.	EAO vs. Cont.	Adj. vs. Cont.
Ct.Th	$\bar{x}$ ± SEM	mm	0.147 ± 0.005	0.155 ± 0.005	0.210 ± 0.007	1.000	0.000	0.000
	Q_1	mm	0.138	0.143	0.189			
	Q_2 (IQR)	mm	0.152 (0.016)	0.157 (0.025)	0.214 (0.034)			
	Q_3	mm	0.154	0.168	0.223			
Ct.S	$\bar{x}$ ± SEM	mm^2	10.538 ± 0.163	9.530 ± 0.238	10.270 ± 0.235	0.026	1.000	0.087
	Q_1	mm^2	10.215	8.917	9.866			
	Q_2 (IQR)	mm^2	10.552 (0.639)	9.560 (1.216)	10.307 (0.896)			
	Q_3	mm^2	10.854	10.133	10.762			
Ct.S/Ct.V	$\bar{x}$ ± SEM	mm^{-1}	18.588 ± 0.608	17.594 ± 0.549	13.670 ± 0.326	0.553	0.000	0.000
	Q_1	mm^{-1}	17.806	16.314	13.098			
	Q_2 (IQR)	mm^{-1}	17.812 (1.953)	17.361 (2.584)	13.553 (1.728)			
	Q_3	mm^{-1}	19.759	18.898	14.825			
BMD	$\bar{x}$ ± SEM	g/cm^3	1.164 ± 0.010	1.270 ± 0.045	1.280 ± 0.006	0.163	0.000	0.993
	Q_1	g/cm^3	1.147	1.207	1.272			
	Q_2 (IQR)	g/cm^3	1.168 (0.032)	1.232 (0.110)	1.287 (0.021)			
	Q_3	g/cm^3	1.180	1.317	1.293			
TMD	$\bar{x}$ ± SEM	g/cm^3	1.480 ± 0.004	1.531 ± 0.015	1.537 ± 0.005	0.053	0.000	0.974
	Q_1	g/cm^3	1.473	1.496	1.526			
	Q_2 (IQR)	g/cm^3	1.478 (0.016)	1.547 (0.064)	1.535 (0.025)			
	Q_3	g/cm^3	1.489	1.559	1.551			

Table S3. Detailed results regarding µCT investigation of distal diaphyseal femur cortex. Measurement of cortical thickness (Ct.Th), cortical surface (Ct.S), cortical surface/volume ratio (Ct.S/Ct.V), bone mineral density (BMD) and tissue mineral density (TMD). Mice were immunized with testicular homogenate in adjuvant (EAO), adjuvant alone (adjuvant) or remained untreated (control). Data represent mean ($\bar{x}$) ± standard error of mean (SEM), lower quartile (Q_1), median quartile (Q_2), upper quartile (Q_3) and interquartile range (IQR).

Parameter	Statistic	Unit	EAO (n = 5)	Adjuvant (n = 6)	Control (n = 7)	p-values EAO vs. Adj.	EAO vs. Cont.	Adj. vs. Cont.
Ct.Th	$\bar{x}$ ± SEM	mm	0.113 ± 0.004	0.125 ± 0.005	0.165 ± 0.002	0.114	0.000	0.000
	Q_1	mm	0.105	0.113	0.159			
	Q_2 (IQR)	mm	0.111 (0.017)	0.127 (0.023)	0.167 (0.009)			
	Q_3	mm	0.122	0.136	0.168			
Ct.S	$\bar{x}$ ± SEM	mm^2	13.012 ± 0.274	11.556 ± 0.326	12.686 ± 0.438	0.059	1.000	0.131
	Q_1	mm^2	12.449	10.745	11.704			
	Q_2 (IQR)	mm^2	13.058 (1.104)	11.787 (1.459)	13.138 (1.602)			
	Q_3	mm^2	13.553	12.204	13.305			
Ct.S/Ct.V	$\bar{x}$ ± SEM	mm^{-1}	24.713 ± 0.982	22.396 ± 0.915	17.706 ± 0.152	0.268	0.004	0.009
	Q_1	mm^{-1}	22.662	20.616	17.536			
	Q_2 (IQR)	mm^{-1}	25.291 (4.063)	21.724 (3.841)	17.605 (0.324)			
	Q_3	mm^{-1}	26.725	24.456	17.860			
BMD	$\bar{x}$ ± SEM	g/cm^3	0.944 ± 0.015	0.993 ± 0.020	1.055 ± 0.005	0.099	0.000	0.015
	Q_1	g/cm^3	0.911	0.972	1.047			
	Q_2 (IQR)	g/cm^3	0.943 (0.068)	1.004 (0.053)	1.058 (0.015)			
	Q_3	g/cm^3	0.979	1.025	1.063			
TMD	$\bar{x}$ ± SEM	g/cm^3	1.273 ± 0.012	1.309 ± 0.017	1.319 ± 0.005	0.165	0.047	1.000
	Q_1	g/cm^3	1.246	1.278	1.310			
	Q_2 (IQR)	g/cm^3	1.286 (0.048)	1.324 (0.062)	1.320 (0.013)			
	Q_3	g/cm^3	1.294	1.340	1.323			

Table S4. Detailed results regarding µCT investigation of the trabecular region in femora. Measurement of trabecular number (Tb.N), trabecular thickness (Tb.Th), trabecular separation (Tb.Sp), bone volume fraction (BV/TV), bone surface density (BS/TV), specific bone surface (BS/BV), structure model index (SMI), trabecular pattern factor (Tb.Pf), bone mineral density (BMD) and tissue mineral density (TMD). Mice were immunized with testicular homogenate in adjuvant (EAO), adjuvant alone (adjuvant) or remained untreated (control). Data represent mean ($\bar{x}$) ± standard error of mean (SEM), lower quartile (Q_1), median quartile (Q_2), upper quartile (Q_3) and interquartile range (IQR).

Parameter	Statistic	Unit	EAO (n = 5)	Adjuvant (n = 6)	Control (n = 7)	p-values EAO vs. Adj.	EAO vs. Cont.	Adj. vs. Cont.
Tb.N	$\bar{x}$ ± SEM	mm^{-1}	1.171 ± 0.099	1.344 ± 0.133	2.771 ± 0.197	1.000	0.000	0.000
	Q_1	mm^{-1}	0.975	1.049	2.558			
	Q_2 (IQR)	mm^{-1}	1.136 (0.411)	1.323 (0.540)	2.962 (0.615)			
	Q_3	mm^{-1}	1.386	1.589	3.173			
Tb.Th	$\bar{x}$ ± SEM	mm	0.038 ± 0.003	0.038 ± 0.002	0.048 ± 0.002	1.000	0.032	0.015
	Q_1	mm	0.032	0.034	0.041			
	Q_2 (IQR)	mm	0.037 (0.013)	0.038 (0.006)	0.048 (0.011)			
	Q_3	mm	0.045	0.040	0.052			
Tb.Sp	$\bar{x}$ ± SEM	mm	0.276 ± 0.006	0.256 ± 0.005	0.199 ± 0.006	0.090	0.000	0.000
	Q_1	mm	0.264	0.248	0.190			
	Q_2 (IQR)	mm	0.275 (0.025)	0.255 (0.018)	0.192 (0.015)			
	Q_3	mm	0.289	0.266	0.206			
BV/TV	$\bar{x}$ ± SEM	%	4.452 ± 0.447	5.065 ± 0.566	13.340 ± 1.283	0.782	0.001	0.001
	Q_1	%	3.509	3.691	10.098			
	Q_2 (IQR)	%	4.254 (1.984)	4.932 (2.524)	15.006 (5.367)			
	Q_3	%	5.493	6.216	15.465			
BS/TV	$\bar{x}$ ± SEM	mm^{-1}	4.427 ± 0.335	5.171 ± 0.440	10.340 ± 0.698	1.000	0.000	0.000
	Q_1	mm^{-1}	3.781	4.186	9.512			
	Q_2 (IQR)	mm^{-1}	4.174 (1.418)	5.098 (1.756)	11.047 (2.197)			
	Q_3	mm^{-1}	5.199	5.942	11.708			
BS/BV	$\bar{x}$ ± SEM	mm^{-1}	101.214 ± 6.639	103.878 ± 4.293	79.430 ± 3.681	1.000	0.017	0.005
	Q_1	mm^{-1}	86.951	94.824	71.430			
	Q_2 (IQR)	mm^{-1}	99.656 (29.306)	102.082 (20.633)	77.165 (20.493)			
	Q_3	mm^{-1}	116.257	115.457	91.923			
SMI	$\bar{x}$ ± SEM		2.252 ± 0.087	2.231 ± 0.100	1.699 ± 0.102	1.000	0.004	0.004
	Q_1		2.112	2.004	1.499			
	Q_2 (IQR)		2.212 (0.299)	2.227 (0.466)	1.585 (0.348)			
	Q_3		2.411	2.469	1.847			
Tb.Pf	$\bar{x}$ ± SEM	mm^{-1}	37.822 ± 2.271	38.736 ± 2.703	22.782 ± 2.360	1.000	0.002	0.001
	Q_1	mm^{-1}	33.323	32.496	18.864			
	Q_2 (IQR)	mm^{-1}	36.131 (9.843)	37.852 (12.877)	20.286 (10.133)			
	Q_3	mm^{-1}	43.166	45.373	28.997			
BMD	$\bar{x}$ ± SEM	g/cm^3	0.078 ± 0.006	0.091 ± 0.008	0.205 ± 0.016	1.000	0.000	0.000
	Q_1	g/cm^3	0.067	0.074	0.164			
	Q_2 (IQR)	g/cm^3	0.073 (0.024)	0.087 (0.035)	0.226 (0.069)			
	Q_3	g/cm^3	0.091	0.110	0.233			
TMD	$\bar{x}$ ± SEM	g/cm^3	0.922 ± 0.018	0.931 ± 0.012	1.002 ± 0.012	1.000	0.003	0.004
	Q_1	g/cm^3	0.883	0.911	0.966			
	Q_2 (IQR)	g/cm^3	0.933 (0.073)	0.939 (0.042)	1.016 (0.059)			
	Q_3	g/cm^3	0.956	0.953	1.025			

Table S5. Detailed results regarding µCT investigation of the trabecular region in vertebral bodies L1. Measurement of trabecular number (Tb.N), trabecular thickness (Tb.Th), trabecular separation (Tb.Sp), bone volume fraction (BV/TV), bone surface density (BS/TV), specific bone surface (BS/BV), structure model index (SMI), trabecular pattern factor (Tb.Pf), bone mineral density (BMD) and tissue mineral density (TMD). Mice were immunized with testicular homogenate in adjuvant (EAO), adjuvant alone (adjuvant) or remained untreated (control). Data represent mean ($\bar{x}$) ± standard error of mean (SEM), lower quartile (Q_1), median quartile (Q_2), upper quartile (Q_3) and interquartile range (IQR).

Parameter	Statistic	Unit	EAO (n = 5)	Adjuvant (n = 6)	Control (n = 7)	p-values EAO vs. Adj.	p-values EAO vs. Cont.	p-values Adj. vs. Cont.
Tb.N	$\bar{x}$ ± SEM	mm^{-1}	3.488 ± 0.215	4.131 ± 0.123	5.243 ± 0.176	0.072	0.000	0.001
	Q_1	mm^{-1}	3.046	3.900	4.917			
	Q_2 (IQR)	mm^{-1}	3.496 (0.881)	4.137 (0.438)	5.187 (0.667)			
	Q_3	mm^{-1}	3.927	4.338	5.584			
Tb.Th	$\bar{x}$ ± SEM	mm	0.038 ± 0.001	0.041 ± 0.001	0.046 ± 0.001	0.044	0.000	0.000
	Q_1	mm	0.036	0.039	0.044			
	Q_2 (IQR)	mm	0.038 (0.003)	0.041 (0.002)	0.046 (0.004)			
	Q_3	mm	0.039	0.042	0.048			
Tb.Sp	$\bar{x}$ ± SEM	mm	0.179 ± 0.008	0.164 ± 0.003	0.147 ± 0.006	0.318	0.004	0.113
	Q_1	mm	0.163	0.157	0.133			
	Q_2 (IQR)	mm	0.177 (0.031)	0.166 (0.013)	0.143 (0.020)			
	Q_3	mm	0.195	0.170	0.154			
BV/TV	$\bar{x}$ ± SEM	%	13.154 ± 0.711	16.856 ± 0.628	24.056 ± 1.132	0.011	0.000	0.001
	Q_1	%	11.502	15.845	21.505			
	Q_2 (IQR)	%	13.661 (3.050)	16.937 (1.946)	25.229 (4.704)			
	Q_3	%	14.552	17.791	26.210			
BS/TV	$\bar{x}$ ± SEM	mm^{-1}	11.951 ± 0.659	13.868 ± 0.342	17.149 ± 0.535	0.075	0.000	0.001
	Q_1	mm^{-1}	10.545	13.127	16.306			
	Q_2 (IQR)	mm^{-1}	12.180 (2.699)	13.877 (1.546)	17.211 (1.936)			
	Q_3	mm^{-1}	13.244	14.673	18.242			
BS/BV	$\bar{x}$ ± SEM	mm^{-1}	90.882 ± 1.376	82.504 ± 1.489	71.647 ± 1.474	0.005	0.000	0.000
	Q_1	mm^{-1}	88.529	78.678	68.219			
	Q_2 (IQR)	mm^{-1}	89.346 (5.474)	83.198 (6.381)	70.618 (7.434)			
	Q_3	mm^{-1}	94.003	85.059	75.653			
SMI	$\bar{x}$ ± SEM		1.400 ± 0.033	1.188 ± 0.054	0.876 ± 0.061	0.056	0.000	0.002
	Q_1		1.321	1.095	0.719			
	Q_2 (IQR)		1.437 (0.139)	1.189 (0.217)	0.887 (0.286)			
	Q_3		1.460	1.313	1.005			
Tb.Pf	$\bar{x}$ ± SEM	mm^{-1}	21.194 ± 0.562	16.390 ± 1.011	10.544 ± 0.927	0.008	0.000	0.001
	Q_1	mm^{-1}	20.020	14.367	8.121			
	Q_2 (IQR)	mm^{-1}	21.353 (2.270)	16.495 (4.232)	10.081 (4.549)			
	Q_3	mm^{-1}	22.290	18.600	12.670			
BMD	$\bar{x}$ ± SEM	g/cm^3	0.173 ± 0.009	0.216 ± 0.011	0.308 ± 0.014	0.080	0.000	0.000
	Q_1	g/cm^3	0.152	0.197	0.282			
	Q_2 (IQR)	g/cm^3	0.180 (0.037)	0.210 (0.038)	0.321 (0.044)			
	Q_3	g/cm^3	0.190	0.235	0.326			
TMD	$\bar{x}$ ± SEM	g/cm^3	0.883 ± 0.004	0.908 ± 0.012	0.995 ± 0.008	0.245	0.000	0.000
	Q_1	g/cm^3	0.872	0.874	0.980			
	Q_2 (IQR)	g/cm^3	0.889 (0.018)	0.915 (0.058)	0.981 (0.027)			
	Q_3	g/cm^3	0.890	0.932	1.007			

Table S6. Detailed results of parameters regarding osteoblasts. Histomorphometrical calculation of relative ALP perimeter (ALP.Pm/Tb.Pm) in enzyme histochemical stained vertebral bodies L3. mRNA expression of ALP and collagen 1α1 (Col1α1) in real-time RT-PCR of vertebral bodies Th10 (normalized to mRNA expression of β-actin). Histomorphometrical calculation of relative osteoid area (O.Ar/B.Ar) in vertebral bodies L2 by Von Kossa/Van Gieson staining. Mice were immunized with testicular homogenate in adjuvant (EAO), adjuvant alone (adjuvant) or remained untreated (control). Data represent mean ($\bar{x}$) ± standard error of mean (SEM), lower quartile (Q_1), median quartile (Q_2), upper quartile (Q_3) and interquartile range (IQR).

Parameter	Statistic	Unit	EAO	Adjuvant	Control	p-values		
						EAO vs. Adj.	EAO vs. Cont.	Adj. vs. Cont.
ALP.Pm/ Tb.Pm	n		5	6	6			
	$\bar{x}$ ± SEM	%	12.720 ± 1.841	21.298 ± 3.237	43.185 ± 4.818	0.386	0.000	0.002
	Q_1	%	9.432	13.026	34.742			
	Q_2 (IQR)	%	10.779 (7.546)	20.910 (16.508)	41.343 (15.548)			
	Q_3	%	16.978	29.534	50.290			
ALP	n		5	6	7			
	$\bar{x}$ ± SEM	−ΔCP	−9.034 ± 0.102	−9.017 ± 0.106	−8.461 ± 0.110	1.000	0.007	0.006
	Q_1	−ΔCP	−9.225	−9.315	−8.760			
	Q_2 (IQR)	−ΔCP	−9.020 (0.375)	−8.955 (0.532)	−8.420 (0.590)			
	Q_3	−ΔCP	−8.850	−8.783	−8.170			
Col1α1	n		5	6	7			
	$\bar{x}$ ± SEM	−ΔCP	−2.092 ± 0.256	−2.490 ± 0.082	−2.411 ± 0.143	0.357	0.570	1.000
	Q_1	−ΔCP	−2.520	−2.620	−2.810			
	Q_2 (IQR)	−ΔCP	−1.940 (0.780)	−2.515 (0.328)	−2.350 (0.700)			
	Q_3	−ΔCP	−1.740	−2.293	−2.110			
O.Ar/B.Ar	n		4	5	7			
	$\bar{x}$ ± SEM	%	21.674 ± 1.662	17.720 ± 4.299	15.695 ± 3.673	1.000	0.469	0.897
	Q_1	%	18.633	10.001	3.348			
	Q_2 (IQR)	%	21.359 (6.397)	17.874 (15.361)	19.556 (19.873)			
	Q_3	%	25.030	25.362	23.221			

Table S7. Detailed results of parameters regarding osteoclasts. Histomorphometrical calculation of number of TRAP positive cells per trabecular perimeter (TRAP.N/Tb.Pm) in enzyme histochemical stained vertebral bodies L3. mRNA expression of cathepsin K (CtsK), receptor activator of nuclear factor-kappa B ligand (RANKL) and osteoprotegerin (OPG) in real-time RT-PCR of vertebral bodies Th10 (normalized to mRNA expression of β-actin). Mice were immunized with testicular homogenate in adjuvant (EAO), adjuvant alone (adjuvant) or remained untreated (control). Data represent mean ($\bar{x}$) ± standard error of mean (SEM), lower quartile (Q_1), median quartile (Q_2), upper quartile (Q_3) and interquartile range (IQR).

Parameter	Statistic	Unit	EAO	Adjuvant	Control	p-values		
						EAO vs. Adj.	EAO vs. Cont.	Adj. vs. Cont.
TRAP.N/	n		5	6	6			
Tb.Pm	$\bar{x}$ ± SEM	n/mm	1.924 ± 0.488	1.933 ± 0.407	5.447 ± 0.527	1.000	0.000	0.000
	Q_1	n/mm	1.048	1.049	4.729			
	Q_2 (IQR)	n/mm	1.508 (1.957)	1.817 (1.859)	5.649 (1.634)			
	Q_3	n/mm	3.005	2.908	6.363			
CtsK	n		5	6	7			
	$\bar{x}$ ± SEM	−ΔCP	−4.522 ± 0.292	−4.097 ± 0.129	−3.754 ± 0.159	0.458	0.039	0.620
	Q_1	−ΔCP	−5.065	−4.370	−4.070			
	Q_2 (IQR)	−ΔCP	−4.310 (0.980)	−4.085 (0.523)	−3.860 (0.640)			
	Q_3	−ΔCP	−4.085	−3.848	−3.430			
RANKL	n		5	6	7			
	$\bar{x}$ ± SEM	−ΔCP	−11.292 ± 0.088	−11.192 ± 0.085	−10.816 ± 0.237	0.797	0.248	0.412
	Q_1	−ΔCP	−11.470	−11.385	−11.420			
	Q_2 (IQR)	−ΔCP	−11.340 (0.380)	−11.200 (0.368)	−11.090 (1.240)			
	Q_3	−ΔCP	−11.090	−11.018	−10.180			
OPG	n		5	6	7			
	$\bar{x}$ ± SEM	−ΔCP	−10.262 ± 0.249	−10.530 ± 0.186	−9.493 ± 0.194	1.000	0.062	0.007
	Q_1	−ΔCP	−10.765	−10.920	−9.770			
	Q_2 (IQR)	−ΔCP	−10.360 (1.055)	−10.650 (0.820)	−9.280 (0.650)			
	Q_3	−ΔCP	−9.710	−10.100	−9.120			

Table S8. Detailed results of parameters regarding osteocytes and cell-contacts. mRNA expression of sclerostin (SOST) and connexin 43 (Cx43) in real-time RT-PCR of vertebral bodies Th10 (normalized to mRNA expression of β-actin). Mice were immunized with testicular homogenate in adjuvant (EAO), adjuvant alone (adjuvant) or remained untreated (control). Data represent mean ($\bar{x}$) ± standard error of mean (SEM), lower quartile (Q_1), median quartile (Q_2), upper quartile (Q_3) and interquartile range (IQR).

Parameter	Statistic	Unit	EAO (n = 5)	Adjuvant (n = 6)	Control (n = 7)	p-values		
						EAO vs. Adj.	EAO vs. Cont.	Adj. vs. Cont.
SOST	$\bar{x}$ ± SEM	−ΔCP	−12.782 ± 0.277	−12.522 ± 0.281	−11.359 ± 0.295	1.000	0.011	0.030
	Q_1	−ΔCP	−13.370	−13.130	−11.970			
	Q_2 (IQR)	−ΔCP	−12.780 (1.175)	−12.450 (1.133)	−11.510 (1.200)			
	Q_3	−ΔCP	−12.195	−11.998	−10.770			
Cx43	$\bar{x}$ ± SEM	−ΔCP	−8.530 ± 0.164	−8.100 ± 0.097	−7.759 ± 0.122	0.111	0.002	0.199
	Q_1	−ΔCP	−8.840	−8.210	−8.100			
	Q_2 (IQR)	−ΔCP	−8.450 (0.580)	−8.060 (0.240)	−7.860 (0.640)			
	Q_3	−ΔCP	−8.260	−7.970	−7.460			

Table S9. Detailed results regarding biomechanical properties of femora in the three-point bending test. Measurement of maximum load, stiffness, post yield displacement and work to fracture. Mice were immunized with testicular homogenate in adjuvant (EAO), adjuvant alone (adjuvant) or remained untreated (control). Data represent mean ($\bar{x}$) ± standard error of mean (SEM), lower quartile (Q_1), median quartile (Q_2), upper quartile (Q_3) and interquartile range (IQR).

						p-values		
Parameter	Statistic	Unit	EAO (n = 4)	Adjuvant (n = 6)	Control (n = 7)	EAO vs. Adj.	EAO vs. Cont.	Adj. vs. Cont.
Maximum Load	$\bar{x}$ ± SEM	N	8.234 ± 1.099	10.392 ± 1.086	15.984 ± 0.665	0.459	0.000	0.001
	Q_1	N	6.106	7.471	14.255			
	Q_2 (IQR)	N	8.342 (4.147)	10.782 (5.094)	16.496 (3.651)			
	Q_3	N	10.253	12.564	17.906			
Stiffness	$\bar{x}$ ± SEM	N/mm	39.331 ± 7.355	45.886 ± 8.284	66.980 ± 4.208	1.000	0.026	0.055
	Q_1	N/mm	30.223	30.375	61.076			
	Q_2 (IQR)	N/mm	33.114 (24.434)	39.360 (30.169)	67.440 (18.950)			
	Q_3	N/mm	54.657	60.544	80.026			
Post Yield Displace-ment	$\bar{x}$ ± SEM	mm	0.982 ± 0.430	0.746 ± 0.151	0.968 ± 0.123	0.933	1.000	0.605
	Q_1	mm	0.169	0.492	0.740			
	Q_2 (IQR)	mm	1.006 (1.602)	0.607 (0.489)	0.950 (0.618)			
	Q_3	mm	1.771	0.981	1.358			
Work To Fracture	$\bar{x}$ ± SEM	N*mm	4.277 ± 1.421	5.566 ± 0.390	12.520 ± 1.380	1.000	0.001	0.001
	Q_1	N*mm	1.756	4.777	9.311			
	Q_2 (IQR)	N*mm	3.958 (5.363)	5.398 (1.453)	12.255 (5.189)			
	Q_3	N*mm	7.119	6.230	14.500			

Table S10. Primer pairs used for real-time RT-PCR.

**Primer**		**Sequence**	**Length [bp[10]]**	**GenBank ID (accession)**
ALP[1]	for[2]	TCA GCT AAT GCA CAA TAT CAA GG	87	NM_007431
	rev[3]	TCC ACA TCA GTT CTG TTC TTC G		
Col1α1[4]	for	TGG CAT CCC TGG ACA GCC TG	144	NM_007742
	rev	ATG GGG CCA GGC ACG GAA AC		
CtsK[5]	for	GAG GCG GCT ATA TGA CCA CT	119	NM_007802
	rev	CTT TGC CGT GGC GTT ATA CA		
RANKL[6]	for	TCC TGT ACT TTC GAG CGC AG	136	NM_011613
	rev	TCA GGT AGT GTG TCT TCA CTC TC		
OPG[7]	for	ACT TCA TCG AAA GCA CCC TGT	181	NM_008764
	rev	TGG TAG GAA CAG CAA ACC TGA		
SOST[8]	for	GCC TCC TCC TGA GAA CAA CC	143	NM_024449
	rev	GGC ATG GGC CGT CTG TC		
Cx43[9]	for	TGC TTC CTC TCA CGT CCC AC	127	NM_010288
	rev	CGC GAT CCT TAA CGC CCT TG		
β-actin	for	TGT TAC CAA CTG GGA CGA CA	165	NM_007393
	rev	GGG GTG TTG AAG GTC TCA AA		

Abbreviations: [1] ALP: alkaline phosphatase, [2] for: forward, [3] rev: reverse, [4] Col1α1: collagen 1α1, [5] CtsK: cathepsin K, [6] RANKL: receptor activator of nuclear factor-kappa B ligand, [7] OPG: osteoprotegerin, [8] SOST: sclerostin, [9] Cx43: connexin 43, [10] bp: base pairs.
